# A tight control of *Rif1* by Oct4 and Smad3 is critical for mouse embryonic stem cell stability

**DOI:** 10.1038/cddis.2014.551

**Published:** 2015-01-08

**Authors:** P Li, X Ma, I R Adams, P Yuan

**Affiliations:** 1 Li Ka Shing Institute of Health Sciences, The Chinese University of Hong Kong, Hong Kong SAR, China; 2 Department of Chemical Pathology, Stem Cell and Functional Genomics, Li Ka Shing Institute of Health Sciences, The Chinese University of Hong Kong, Hong Kong SAR, China; 3 The Chinese University of Hong Kong Shenzhen Research Institute, Shenzhen, China; 4 MRC Human Genetics Unit, MRC Institute of Genetics and Molecular Medicine, University of Edinburgh, Western General Hospital, Edinburgh, UK; 5 School of Biomedical Sciences, The Chinese University of Hong Kong, Hong Kong SAR, China

## Abstract

Prolonged culture of embryonic stem cells (ESCs) leads them to adopt embryonal carcinoma cell features, creating enormous dangers for their further application. The mechanism involved in ESC stability has not, however, been extensively studied. We previously reported that SMAD family member 3 (Smad3) has an important role in maintaining mouse ESC stability, as depletion of *Smad3* results in cancer cell-like properties in ESCs and *Smad3−/−* ESCs are prone to grow large, malignant teratomas. To understand how Smad3 contributes to ESC stability, we performed microarray analysis to compare the transcriptome of wild-type and *Smad3−/−* ESCs. We found that Rif1 (RAP1-associated protein 1), a factor important for genomic stability, is significantly upregulated in *Smad3−/−* ESCs. The expression level of *Rif1* needs to be tightly controlled in ESCs, as a low level of *Rif1* is associated with ESC differentiation, but a high level of *Rif1* is linked to ESC transformation. In ESCs, Oct4 activates *Rif1*, whereas Smad3 represses its expression. Oct4 recruits Smad3 to bind to *Rif1* promoter, but Smad3 joining facilitates the loading of a polycomb complex that generates a repressive epigenetic modification on *Rif1* promoter, and thus maintains the expression of *Rif1* at a proper level in ESCs. Interestingly, Rif1 short hairpin RNA (shRNA)-transduced *Smad3−/−* ESCs showed less malignant properties than the control shRNA-transduced *Smad3−/−* ESCs, suggesting a critical role of Rif1 in maintaining the stability of ESCs during proliferation.

Embryonic stem cells (ESCs) can serve as a rich source of differentiated cells for cell-based therapy due to their pluripotency and unlimited self-renewal capacity. However, prolonged culture of ESCs results in ESCs accumulating numerous mutations, and they gradually adopt embryonal carcinoma cell features.^1, 2, 3^ This prompts serious safety concerns with regard to ESC applications and also raises important questions regarding how ESCs maintain their genomic stability.

Transforming growth factor beta (TGF-*β*) signaling has an important role in development and homeostasis. It also functions in multiple diseases such as cancer, tissue fibrosis and diabetes.^4, 5^ Through their respective ligand receptors, TGF-*β*/Activin/Nodal activates SMAD family member (Smad)2/Smad3. The activated Smads bind to Smad4 and translocate from cytoplasm to the nucleus to regulate the downstream genes.^6, 7^ TGF-*β*/Activin/Nodal signaling is crucial for maintaining self-renewal and pluripotency in human ESCs, but appears to be dispensable for the pluripotency of mouse ESCs.^8, 9^ Instead, the activation of Activin/Nodal signaling is required for the propagation of mouse ESCs.^10, 11^ Smad3 is a downstream factor of TGF-*β*/Activin/Nodal signaling. Although depletion of *Smad3* leads to transient expression distortion of mesoderm markers during embryoid body (EB) formation, the final lineage formation is not affected,^12^ as *Smad3* knockout mice are viable and fertile.^13^ This may be because Smad2, another downstream factor of TGF-*β*/Activin/Nodal signaling, has a redundant role of Smad3. It has been reported that Smad2 and Smad3 collaboratively regulate mesoderm formation during embryo development.^12, 14^ Previously, we found that activation of Smad3 is vitally important for ESCs to maintain their genetic integrity during propagation, as depletion of *Smad3* leads mouse ESCs to adopt cancer cell properties.^12^ To further illustrate how Smad3 contributes to ESC stability, we performed microarray assay to identify genes that show an obvious change after *Smad3* depletion. Among the genes affected by *Smad3* depletion, Rif1 (RAP1-associated protein 1), a factor closely associated with chromatin stability, shows the greatest upregulation.

Rif1 is first identified in budding yeast as a Rap1-interacting factor. It is recruited to the telomere by Rap1 and implicated in maintaining telomere structure and homeostasis.^15, 16^ In mammalian cells, except the regulation of telomere homeostasis,^17, 18^ Rif1 mediates the ATM (ataxia telangiectasia mutated)/53BP1 (tumor suppressor p53-binding protein 1) signaling after DNA damage to repress break resection and promote the non-homologous end joining (NHEJ) mechanism in G1 phase.^19, 20, 21, 22, 23^ In addition, Rif1 globally regulates the replication-timing program in both yeast fission and mammalian cells.^24, 25, 26, 27^ Rif1 localizes to the stalled replication forks in response to ATR activation and serves as a component of the DNA replication checkpoint.^28, 29, 30, 31^ Rif1 is also highly expressed in the pluripotent stem cells.^32, 33, 34^ Knockdown of *Rif1* by RNA interference in mouse ESCs leads to ESC differentiation.^35^

In this study, we determine that Rif1 is an important contributor to ESC stability during its propagation. Rif1 expression level is tightly controlled by Smad3 and Oct4. Reduction of *Rif1* by RNA interference leads *Smad3−/−* ESCs to show less malignant properties than control shRNA knockdown *Smad3−/−* ESCs, suggesting that upregulation of Rif1 is a key factor in the transformation of *Smad3−/−* ESCs.

## Results

### *Rif1* is a direct downstream target of Smad3

**Figure 1:**
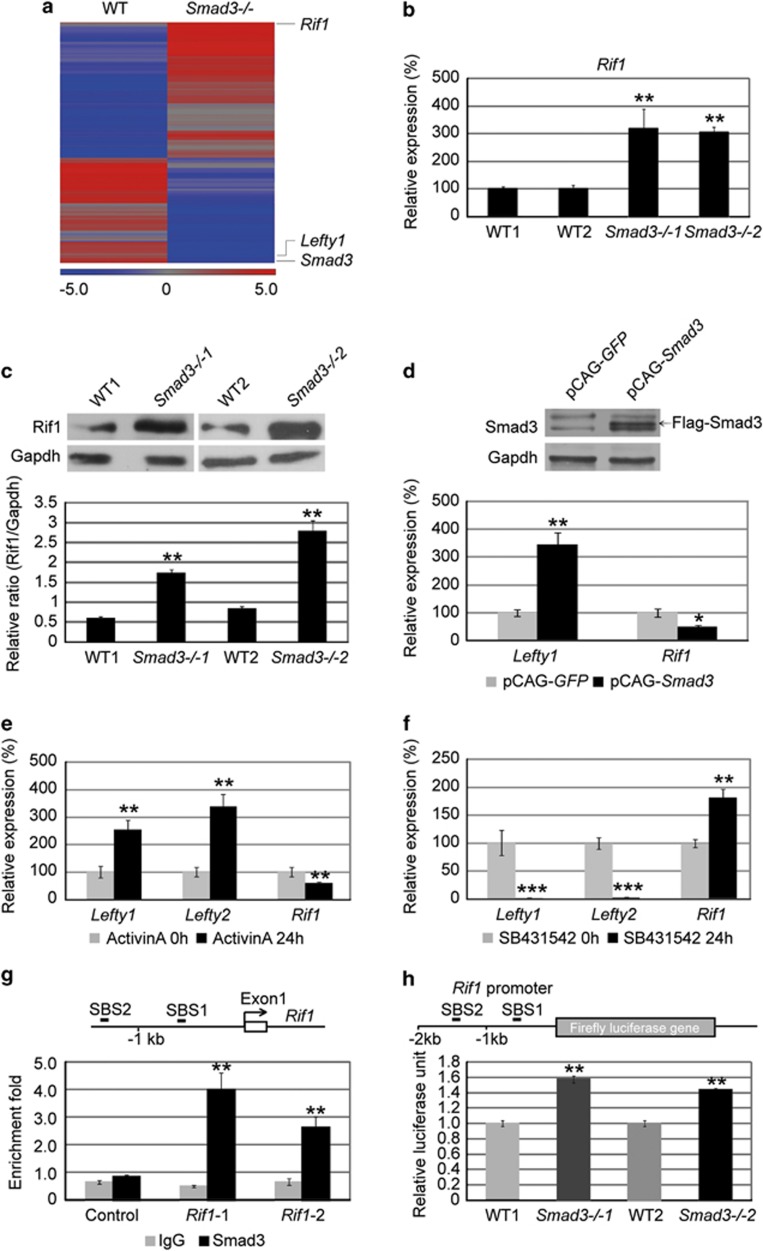
Smad3 represses *Rif1* expression in ESCs. (**a**) The heat map shows the expression profile of genes with mRNA level increased or decreased for more than 1.5-fold in *Smad3−/−* ESCs compared with WT ESCs. *Lefty1* and *Smad3* are decreased, whereas *Rif1* is increased in *Smad3−/−* ESCs compared with WT ESCs. (**b**) Quantitative real-time PCR to examine the mRNA level of *Rif1* in WT and *Smad3−/−* ESCs. *Actin* was analyzed as an internal control. The data are shown as the mean±S.D. (*n*=3). (**c**) Western blot and densitometric analyses of the expression of Rif1 in WT and *Smad3−/−* ESCs. Gapdh expression level was used as an internal control. The data are shown as the mean±S.D. (*n*=2). (**d**) Western blot analysis of Smad3 (upper layer) and real-time PCR analysis of *Lefty1* and *Rif1* (lower layer) in pCAG-*GFP-* and pCAG-*Smad3*-transfected *Smad3−/−* ESCs. Gapdh protein level and *Actin* expression level were used as internal controls for the western blot and real-time PCR analysis, respectively. Arrow indicates the overexpression band of Flag-Smad3. The real-time PCR data are shown as the mean±S.D. (*n*=3). (**e**) Quantitative real-time PCR to examine mRNA expression levels of *Lefty1, Lefty2* and *Rif1* in mouse ESCs with Activin A (25 ng/ml) treatment for 0 and 24 h. *Actin* was analyzed as an internal control. The data are shown as the mean±S.D. (*n*=3). (**f**) Quantitative real-time PCR to examine mRNA expression levels of *Lefty1, Lefty2* and *Rif1* in mouse ESCs with SB431542 (10 *μ*M) treatment for 0 and 24 h. *Actin* was analyzed as an internal control. The data are shown as the mean±S.D. (*n*=3). (**g**) ChIP-qPCR to examine Smad3 and IgG enrichment on the promoter of *Rif1*. The sketch of Smad3-binding sites on the promoter of *Rif1* has been indicated (SBS1 and SBS2), *Rif1*-1 and *Rif1*-2 regions cover SBS1 and SBS2, respectively. Protein enrichment on *Actin* was analyzed as a control. The data are shown as the mean±S.D. (*n*=3). (**h**) Luciferase assay to examine *Rif1* promoter activity in WT and *Smad3−/−* ESCs at 48 h after transfection. Two  kb *Rif1* promoter was cloned in front of firefly luciferase reporter. Renilla was analyzed as an internal control. The data are shown as the mean±S.D. (*n*=3). Statistically significant differences, calculated through student's *t*-tests, are indicated (*P<0.05; **P<0.01; ***P<0.001)

Previously, we reported that depletion of *Smad3* in mouse ESCs produced cancer cell-like features.^12^ To understand the underlying mechanism, cDNA microarray analysis was performed to compare the transcriptome between wild-type (WT) and *Smad3−/−* ESCs. Genes with more than a 1.5-fold difference between WT and *Smad3−/−* ESCs were selected by Partek software to generate a heat map. On the basis of the microarray data, the expression of *Smad3* and *Lefty1* was markedly reduced in *Smad3−/−* ESCs. Besides, validation of eight randomly picked genes by real-time PCR further suggests that the microarray result was reliable. Among the genes that show different expression after *Smad3* depletion, *Rif1* ranked as the highest upregulated gene in *Smad3−/−* ESCs (Figure 1a and Supplementary Figure S1A). Real-time PCR and western blot analysis confirmed the upregulation of Rif1 at both mRNA and protein level in *Smad3−/−* ESCs (Figures 1b and c). Furthermore, overexpression of *Smad3* in *Smad3−/−* ESCs could significantly downregulate *Rif1* expression, but upregulate *Lefty1* expression (Figure 1d). As Smad3 is a downstream factor of the Activin pathway in mouse ESCs,^10^ we treated ESCs with Activin A (25 ng/ml) and Activin A inhibitor SB431542 (10 μM), respectively, to examine the expression of *Rif*1. As expected, the expression of *Rif1* was decreased by Activin A treatment, but increased by SB431542 treatment. The expression of *Lefty1* and *Lefty2* was regulated conversely, confirming that *Lefty1* and *Lefty2* are positively regulated by Activin/Smad3 pathway, whereas *Rif1* is negatively regulated by this pathway (Figures 1e and f). On the basis of Mullen's chromatin immunoprecipitation (ChIP)-seq data, there are two Smad3-binding sites (SBS1 and SBS2) at the promoter region of *Rif1.*^4^ Therefore, we designed primers to quantitate *Rif1-1* and *Rif1-2* regions that cover SBS1 and SBS2, respectively. Examining ChIP-enriched DNA by real-time PCR, we found that Smad3 specifically bound to the *Rif1-1* and *Rif1-2* regions (Figure 1g). To further examine whether *Rif1* promoter activity was affected by *Smad3* depletion, a luciferase assay was performed with the *Rif1* promoter containing the Smad3-binding sites. The result showed that *Rif1* promoter activity was enhanced in *Smad3−/−* ESCs compared with WT ESCs (Figure 1h). Taken together, all these data demonstrated that *Rif1* is a target of the Activin/Smad3 pathway, and that Smad3 represses *Rif1* expression in mouse ESCs.

### Inverse expression profiles between *Smad3* and *Rif1*

To further examine the correlation between *Smad3* and *Rif1*, we examined the expression profiles of these two genes in mouse ESCs, mouse embryonic fibroblasts (MEFs), teratoma cells and mouse ESC-differentiated cells. *Smad3* showed higher expression in MEFs and teratoma cells than in ESCs. Conversely, *Rif1* was expressed at a lower level in MEFs and teratoma cells than in ESCs (Supplementary Figures S1B, S1C and S2A). In addition, we also traced the expression changes of *Smad3* and *Rif1* during ESC differentiation using monolayer culture and EB formation (Supplementary Figures S1D and S2B). ESC differentiation was accompanied by gradual downregulation of pluripotent markers and expression of lineage markers (Supplementary Figures S1E and S2C). During ESC differentiation, the expression of *Smad3* mRNA was gradually increased, whereas the expression of *Rif1* mRNA was decreased (Supplementary Figures S1F and S2D). Interestingly, although the expression of *Rif1* was decreased in *Smad3−/−* ESC-formed EBs, the *Rif1* level was always higher in *Smad3−/−* ESC-formed EBs than in WT ESC-formed EBs at the same stage, suggesting that Smad3 is one of the key components regulating *Rif1* expression during ESC differentiation (Supplementary Figure S2E). These expression profiles also confirm previous reports that Rif1 is a factor associated with pluripotency.^32, 35^

### Oct4 is required for Smad3 to bind to *Rif1* promoter

**Figure 2:**
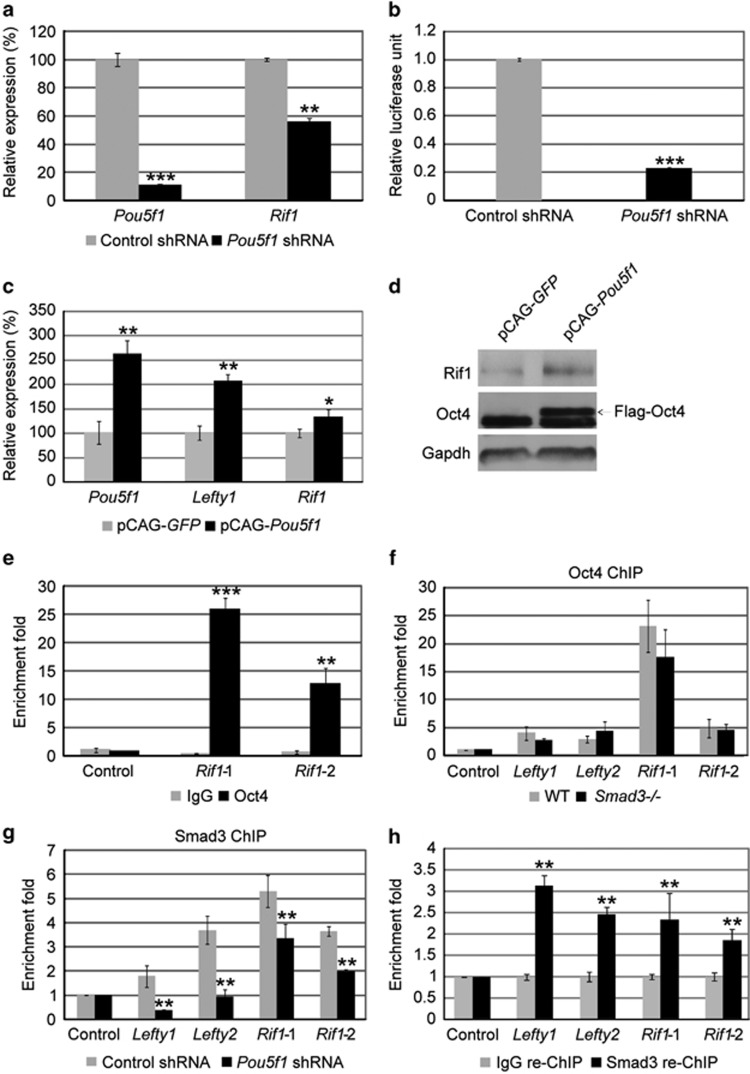
Oct4 positively regulates *Rif1* and is indispensable for Smad3 to bind to *Rif1* promoter regions. (**a**) Quantitative real-time PCR to examine the mRNA levels of *Pou5f1* and *Rif1* after transfection with pSuper control and pSuper-*Pou5f1-*shRNA plasmids. *Actin* was analyzed as a control. The data are shown as the mean±S.D. (*n*=3). (**b**) Luciferase assay to examine *Rif1* promoter activity in mouse ESCs transfected with pSuper control and pSuper-*Pou5f1-*shRNA plasmids. Renilla was analyzed as an internal control. The data are shown as the mean±S.D. (*n*=3). (**c**) Quantitative real-time PCR to examine the mRNA levels of *Pou5f1*, *Lefty1* and *Rif1* after transfection with pCAG-*GFP* and pCAG-*Pou5f1* plasmids. *Actin* was analyzed as a control. The data are shown as the mean±S.D. (*n*=3). (**d**) Western blot analysis of the protein levels of Oct4 and Rif1 in mouse ESCs transfected with pCAG-*GFP* and pCAG-*Pou5f1* plasmids. Gapdh was analyzed as a control. Arrow indicates the overexpression band of Flag-Oct4. (**e**) ChIP-qPCR to examine the DNA enrichment of Oct4 and control IgG at the Smad3-binding sites on the promoter of *Rif*1 in mouse ESCs. Enrichment of studied proteins on *Actin* was analyzed as a control. The data are shown as the mean±S.D. (*n*=3). (**f**) ChIP-qPCR to examine the Oct4 enrichment at the *Lefty1*, *Lefty2* and *Rif1* in WT and *Smad3−/−* ESCs. *Actin* was analyzed as a control. The data are shown as the mean±S.D. (*n*=3). (**g**) ChIP-qPCR to examine Smad3 enrichment at the *Lefty1*, *Lefty2* and *Rif1* at 1-day puromycin selection after mouse ESCs were transfected with pSuper control and pSuper-*Pou5f1*-shRNA plasmids. Smad3 enrichment at *Actin* was analyzed as a control. The data are shown as the mean±S.D. (*n*=3). (**h**) Sequential ChIP assay was performed to examine Smad3 and IgG enrichment on Oct4-enriched DNAs. The quantity of enriched *Lefty1*, *Lefty2* and *Rif1* (*Rif1*-1 and *Rif1*-2) fragments was checked by real-time PCR. The data are shown as the mean±S.D. (*n*=3). Statistically significant differences, calculated through student's *t*-tests, are indicated (*P<0.05; **P<0.01; ***P<0.001)

Multiple Oct4-bound genes are found to be co-occupied by Smad3 and respond to TGF-*β* signaling.^4^ To find out whether *Rif1* is among these genes, we first knocked down the expression of *Pou5f1* by RNA interference. The expression level of *Rif1* was significantly decreased after *Pou5f1* knockdown (Figure 2a). This is consistent with the luciferase activity of *Rif1* promoter being reduced to 20% after *Pou5f1* knockdown, suggesting that *Rif1* is regulated by Oct4 (Figure 2b). Furthermore, overexpression of *Pou5f1* in mouse ESCs can enhance the expression of *Rif1* at both mRNA and protein level (Figures 2c and d). To determine whether Oct4 co-binds with Smad3 on *Rif1* promoter region, we performed ChIP assay with Oct4 antibody. As expected, Oct4 was highly enriched on the promoter regions of *Rif1* where Smad3 binds (Figure 2e). These data support previous reports that Oct4 positively regulates *Rif1* expression in mouse ESCs.^33, 35^

Through co-immunoprecipitation and sequential ChIP experiments, Mullen *et al.*^4^ discovered that Oct4 can form a complex with Smad3 and recruit Smad3 to a *Lefty1* enhancer to regulate *Lefty1* expression in mouse ESCs. Prompted by the opposed regulatory roles of Smad3 and Oct4 on *Rif1*, we first examined whether Smad3 is required for Oct4 to bind to *Rif1* promoter. As the Oct4 protein level is similar in WT and *Smad3−/−* ESCs,^12^ we directly performed a ChIP assay with Oct4 antibody. We found that Oct4 enrichment on *Lefty1*, *Lefty2* and *Rif1* in WT and *Smad3−/−* ESCs was not obviously affected by the depletion of *Smad3* (Figure 2f). We then investigated whether Oct4 is required for recruiting Smad3 to bind to *Rif1*. We knocked down *Pou5f1* by shRNA. After 1-day selection with puromycin, the mRNA level of *Pou5f1* was significantly reduced, but the expression of *Smad3*, pluripotent marker *Nanog*, lineage markers *Cdx2*, *Cxcr4* and *T* were not significantly changed and ESCs still maintained the colony morphology (Supplementary Figure S3A). Meanwhile, the Oct4 protein level was obviously decreased, whereas the Smad3 was not affected (Supplementary Figure S3B). We performed ChIP assay with Smad3 antibody using cells at this stage and found that the binding efficiency of Smad3 on *Lefty1*, *Lefty2* and *Rif1* was significantly reduced after *Pou5f1* knockdown (Figure 2g). To further confirm that Oct4 and Smad3 co-bind to *Rif1* promoter, we performed re-ChIP assay with a Smad3 antibody after Oct4 ChIP and discovered that Smad3 and Oct4 do bind to *Rif1* promoter simultaneously (Figure 2h). These data suggested that Oct4 is required for Smad3 to bind to *Rif1* promoter, but Smad3 is not required for Oct4 binding.

### *Rif1* promoter shows Smad3-dependent H3K27 methylation

**Figure 3:**
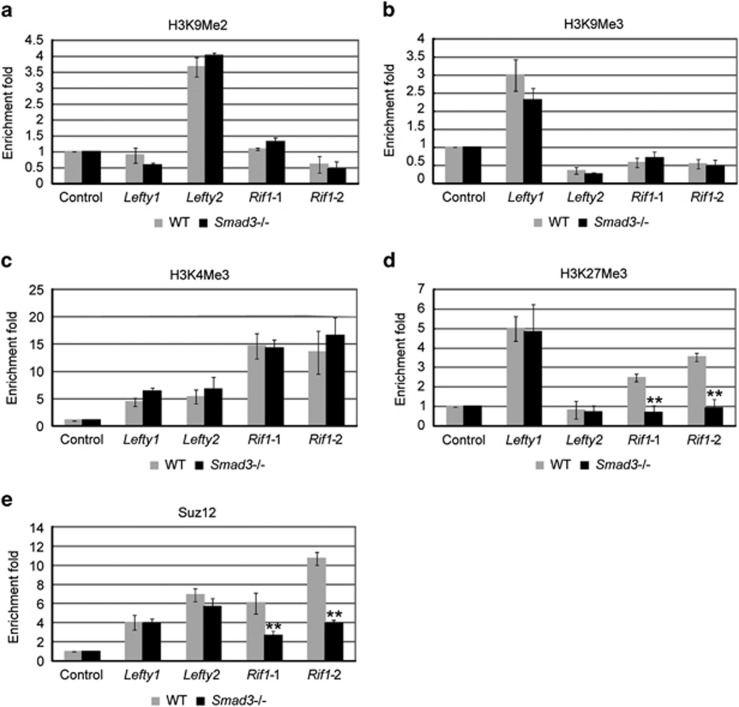
Rif1 promoter shows Smad3-dependent H3K27 methylation ChIP-qPCR to examine (**a**) H3K9me2, (**b**) H3K9me3, (**c**) H3K4Me3, (**d**) H3K27Me3 and (**e**) Suz12 enrichment at *Lefty1* and *Lefty2* enhancer and *Rif1* promoter in WT and *Smad3−/−* ESCs. Enriched *Actin* was analyzed as a negative control. The data are shown as the mean±S.D. (*n*=3). Statistically significant differences, calculated through student's *t*-tests, are indicated (*P<0.05; **P<0.01; ***P<0.001)

Mullen *et al.*^4^ reported that Activin could induce both upregulation and downregulation of the expression of Oct4 and Smad3 co-occupied genes, indicating that Oct4 and Smad3 regulate their targets by sophisticated regulatory mechanisms. To uncover how Oct4 and Smad3 regulate *Rif1*, we performed the ChIP assay with histone modification markers, H3K9me2, H3K9me3, H3K4Me3 and H3K27Me3. H3K9me2 and H3K9me3 label the heterochromatin and H3K4me3 and H3K27me3 are the bivalent markers that label genes related to pluripotency and differentiation.^36, 37, 38^ *Rif1* promoter was enriched by H3K4me3 and H3K27me3, but not by H3K9me2 and H3K9me3 (Figures 3a and d). Depletion of *Smad3* did not affect H3K4me3 enrichment (Figure 3c), but seriously affected H3K27me3 enrichment. However, H3K27me3 level at *Lefty1* and *Lefty2* was not obviously affected after *Smad3* depletion (Figure 3d). This result implies that *Rif1* expression is controlled by a specific epigenetic modification involving Smad3. As Suz12 is the key component of the polycomb repressive complex (PRC2) and contributes to H3K27me3, we performed a ChIP assay with a Suz12 antibody. We found that Suz12 enrichment on *Rif1* is significantly reduced after *Smad3* depletion, but its enrichment on *Lefty1* and *Lefty2* was stable (Figure 3e). Taken together, these data suggest that, although Oct4 and Smad3 co-bind to *Lefty1*, *Lefty2* and *Rif1*, the mechanism used to regulate *Rif1* is different to that used to regulate *Lefty1* and *Lefty2*. In the *Rif1* regulatory complex, Smad3 has a critical role in loading PRC2 to regulate the expression of *Rif1* through H3K27 methylation.

### *Smad3−/−* ESCs show higher cell proliferation and DNA repair capacities than WT ESCs after ultraviolet irradiation

**Figure 4:**
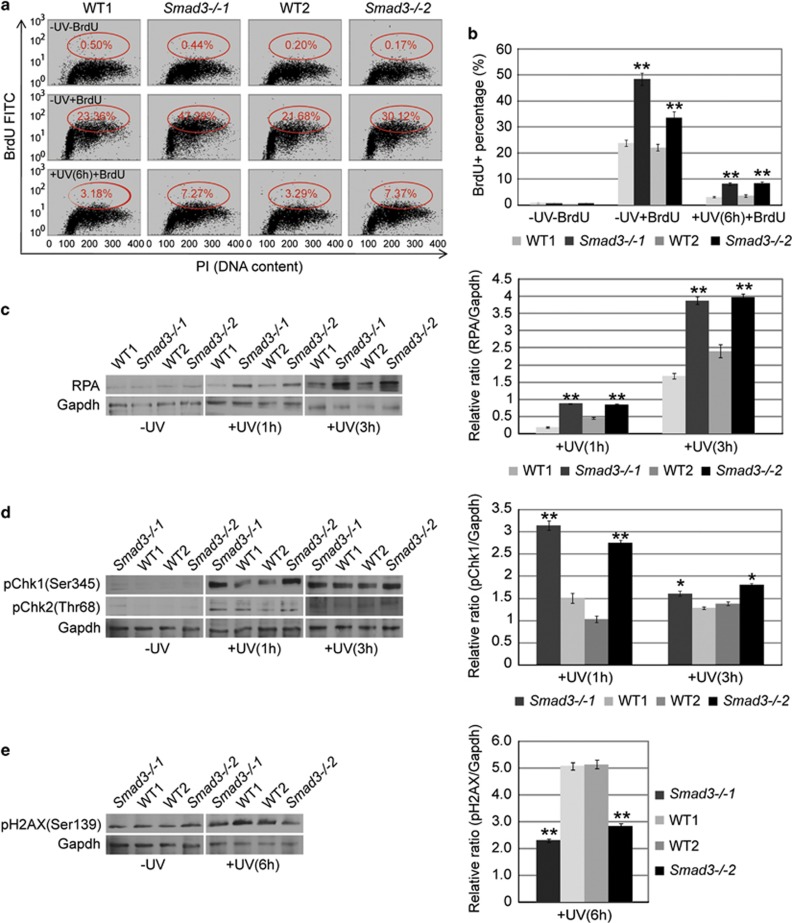
*Smad3−/−* ESCs show enhanced cell proliferation and DNA repair capacity after UV irradiation. (**a**) Representative flow cytometry dot plot of BrdU-integrated WT (WT1 and WT2) and *Smad3−/−* (*Smad3−/−1* and *Smad3−/−2*) ESCs at 0 and 6 h after UV irradiation. *x* axis represents DNA content through PI stain and *y* axis represents BrdU-FITC-labeled cells. (**b**) Statistical analysis of the BrdU-positive cell percentage of **a** from two independent experiments. The data are shown as the mean±S.D. (*n*=2). (**c**) Western blot and densitometric analyses of the expression of RPA2 in WT (WT1 and WT2) and *Smad3−/−* (*Smad3−/−1* and *Smad3−/−2*) ESCs before, and at 1 and 3 h after, UV (40 mJ/cm^2^) irradiation. Gapdh expression level was used as an internal control. The densitometric data are shown as the mean±S.D. (*n*=2). (**d**) Western blot and densitometric analyses of the expression of pChk1 (Ser345) and pChk2 (Thr68) in WT (WT1 and WT2) and *Smad3−/−* (*Smad3−/−1* and *Smad3−/−2*) ESCs before, and at 1 and 3 h after, UV (40 mJ/cm^2^) irradiation. Gapdh expression level was used as an internal control. The densitometric data are shown as the mean±S.D. (*n*=2). (**e**) Western blot and densitometric analyses of the expression of pH2AX (Ser139) in WT (WT1 and WT2) and *Smad3−/−* (*Smad3−/−1* and *Smad3−/−2*) ESCs before, and at 6 h after, UV (40 mJ/cm^2^) irradiation. Gapdh expression level was used as an internal control. The data are shown as the mean±S.D. (*n*=2). Statistically significant differences, calculated through student's *t*-tests, are indicated (*P<0.05; **P<0.01; ***P<0.001)

Our previous studies showed that *Smad3* depletion enhanced the anti-apoptosis capacity of ESCs. To further substantiate this observation, we set out to examine the response of WT and *Smad3−/−* ESCs to DNA damage in detail. Before ultraviolet (UV) irradiation, WT and *Smad3−/−* ESCs showed no obvious difference after propidium iodide (PI) staining. Further, labeling the cells with bromodeoxyuridine (BrdU) revealed that more *Smad3−/−* ESCs showed active DNA replication than WT ESCs. WT and *Smad3−/−* ESCs were then exposed to UV irradiation (40 mJ/cm^2^) to induce DNA damage. Five hours later, the cells were pulse labeled with BrdU and cultured for another 30 min. Subsequently, the cells were collected and stained with PI and FITC-conjugated BrdU antibody for flow cytometry analysis. About 7% *Smad3−/−* ESCs showed active DNA replication compared with only about 3% for the WT cells (Figures 4a and b). This result demonstrated that *Smad3−/−* ESCs have a higher cell proliferation capacity than WT ESCs after DNA damage.

In responding to the DNA damage after UV irradiation, ATR-Chk1 and ATM-Chk2 are activated to modulate checkpoint, DNA repair, apoptosis and cell senescence. Replication protein A (RPA) is an ssDNA-binding protein in eukaryotes and prevents ssDNA from forming hairpin structures or re-annealing when the DNA is under repair, replication or recombination.^39, 40, 41, 42^ RPA is upregulated after DNA damage and it is essential for the ATR-mediated DNA damage checkpoint.^43, 44^ *Smad3−/−* ESCs expressed a significantly higher level of RPA than WT ESCs at 1 h and 3 h after UV irradiation (Figure 4c). Consistent with this, phosphorylated Chk1 was significantly higher in *Smad3−/−* ESCs than WT ESCs, whereas phosphorylated Chk2 was only slightly increased in *Smad3−/−* ESCs (Figure 4d). These data imply an elevated DNA damage response in *Smad3−/−* ESCs.

H2AX, a histone H2A variant, is phosphorylated by ATM after DNA damage. It binds to the damaged DNA and attracts more proteins to join the DNA repair, thus it is a good indicator of DNA damage.^45, 46, 47, 48^ Significant amounts of H2AX were detected in both WT and *Smad3−/−* ESCs 1 h after UV irradiation, suggesting substantial DNA damage in these cells (Supplementary Figure S5A). However, 6 h after UV irradiation, phosphorylated H2AX was significantly less in *Smad3−/−* ESCs than WT ESCs (Figure 4e), indicating that, at this point in time, less-damaged DNA remained in the *Smad3−/−* ESCs. All these observations confirm that *Smad3−/−* ESCs have an enhanced DNA repair ability after UV irradiation. Rif1 has been demonstrated to be highly associated with UV-induced DNA damage response and checkpoint activation.^30, 48^ We analyzed the phosphorylation level of Chk1 in *Smad3−/−* ESCs after being transfected with Rif1 shRNA and treated with UV irradiation. Phosphorylated Chk1 was significantly reduced in the *Rif1* knockdown *Smad3−/−* ESCs compared with the control knockdown *Smad3−/−* ESCs (Supplementary Figure S5B), suggesting that upregulation of *Rif1* in *Smad3−/−* ESCs mediates the enhanced DNA damage response after UV irradiation.

### Reduced cell proliferation by knockdown of *Rif1* in *Smad3−/−* ESCs

**Figure 5:**
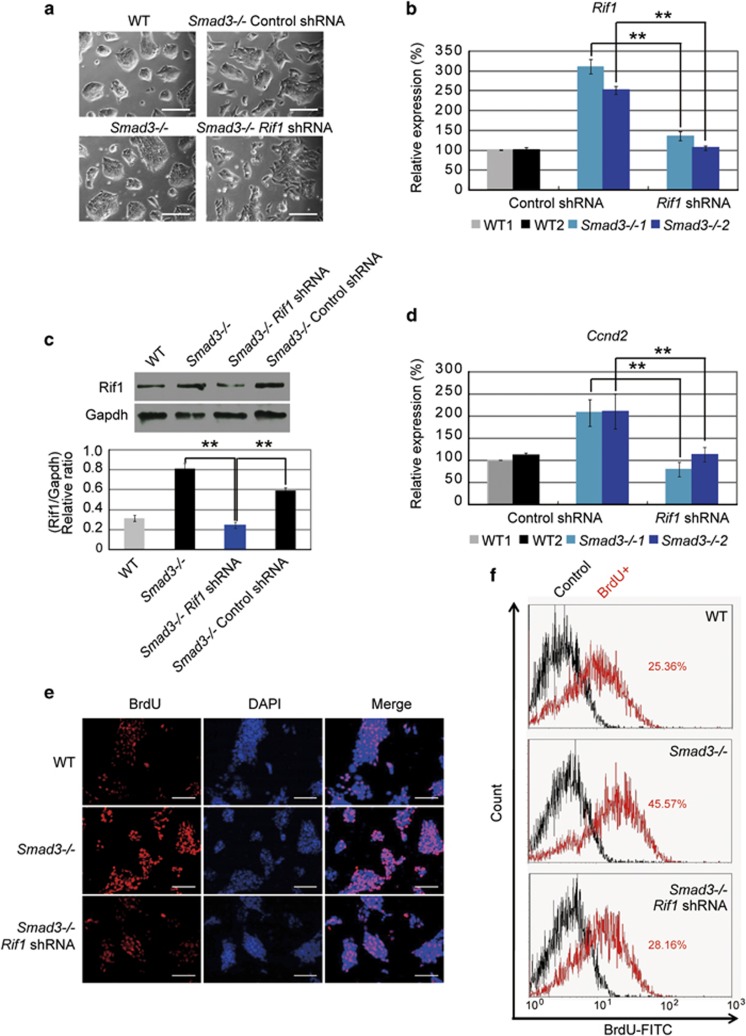
Knockdown of *Rif1* in *Smad3−/−* ESCs can attenuate cell proliferation. (**a**) Morphological appearances of WT ESCs, *Smad3−/−* ESCs and control shRNA- and Rif1 shRNA-transduced *Smad3−/−* ESCs. Scale bar=200 μm. (**b**) Quantitative real-time PCR to examine the mRNA level of *Rif1* in WT ESCs and *Smad3−/−* ESCs after being transfected with control shRNA and Rif1 shRNA plasmids. *Actin* was analyzed as an internal control. The data are shown as the mean±S.D. (*n*=3). (**c**) Western blot and densitometric analyses of the expression of Rif1 in WT, *Smad3−/−* ESCs and control shRNA- and Rif1 shRNA-transduced *Smad3−/−* ESCs. Gapdh expression level was used as an internal control. The data are shown as the mean±S.D. (*n*=2). (**d**) Quantitative real-time PCR to examine the mRNA level of *Ccnd2* in WT ESCs and control shRNA- and Rif1 shRNA-transduced *Smad3−/−* ESCs. *Actin* was analyzed as an internal control. The data are shown as the mean±S.D. (*n*=3). Statistically significant differences, calculated through student's *t*-tests, are indicated (*P<0.05; **P<0.01; ***P<0.001). (**e**) Immunofluorescence staining with BrdU antibody to examine BrdU integration in WT ESCs and control shRNA- and Rif1 shRNA-transduced *Smad3−/−* ESCs. Cells were pulse labeled with BrdU for 30 min, and then fixed for BrdU staining. The nuclei were stained with DAPI. Scale bar=200 μm. (**f**) Flow cytometry analysis of BrdU integration percentage (pulse labeled with 30 min) in WT, control shRNA- and Rif1 shRNA-transduced *Smad3−/−* ESCs. *x* axis represents BrdU-FITC cell percentage and *y* axis represents cell counts

To check whether the upregulation of *Rif1* is a major factor for *Smad3−/−* ESC-enhanced proliferation, two shRNAs targeting to two different regions of *Rif1* gene were constructed. Both of them could efficiently reduce Rif1 at both mRNA and protein level. Knockdown of *Rif1* caused ESC differentiation with downregulation of pluripotent markers alkaline phosphatase (AP) and Oct4 and upregulation of differentiation markers *Cdx2*, *Gata6*, *T* and *Fgf5* (Supplementary Figures S4A–S4D). Next we transfected Rif1 shRNA into the *Smad3−/−* ESCs. We found that the expression level of *Rif1* in *Smad3−/−* ESCs could be reduced to about the *Rif1* level in WT ESCs by Rif1 shRNA after selection with puromycin for 3 days (Figures 5a and b). To constantly reduce *Rif1* expression in *Smad3−/−* ESCs, we transduced *Smad3−/−* ESCs with lentivirus to stably express Rif1 shRNA. By picking a number of single-cell colonies, we were able to select *Smad3−/−* ESCs with stably reduced *Rif1*. Rif1 shRNA-transduced *Smad3−/−* ESCs expressed significantly lower Rif1 than *Smad3−/−* ESCs and control shRNA-transduced *Smad3−/−* ESCs, but a similar level of Rif1 to WT ESCs (Figure 5c). These Rif1 shRNA-transduced *Smad3−/−* ESCs could still proliferate. The expression of *Ccnd2* (cyclin-D2), which is increased in *Smad3−/−* ESCs,^12^ was significantly decreased by *Rif1* reduction (Figure 5d). Besides, BrdU integration assay revealed that proliferating cell number in *Rif1* knockdown *Smad3−/−* ESCs was significantly reduced to about the WT ESC level (Figures 5e and f), suggesting that upregulation of *Rif1* in *Smad3−/−* ESCs is one of the main factors enhancing ESC proliferation.

### The cell migration capacity of *Smad3−/−* ESC-differentiated cells is reduced by knockdown of *Rif1*

**Figure 6:**
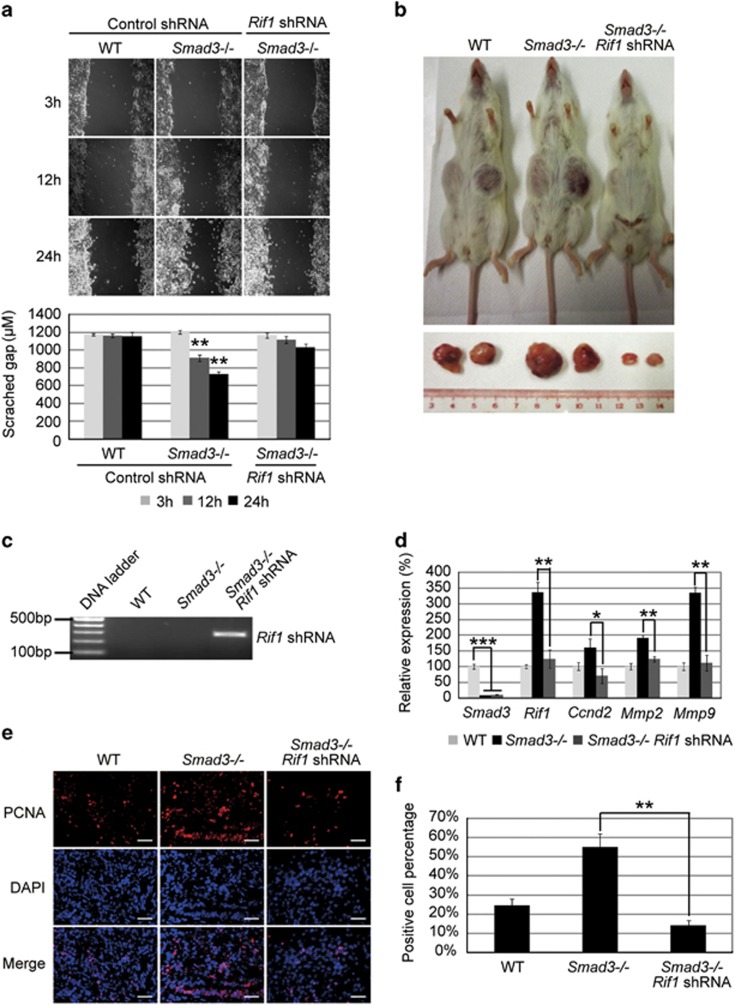
Knockdown of *Rif1* in *Smad3−/−* ESCs can attenuate cell migration. (**a**) Image (upper) and histogram (lower) of scratch wound healing of WT ESCs and control shRNA- and Rif1 shRNA-transduced *Smad3−/−* ESC-differentiated cells at 3, 12 and 24 h after scratch. The data are shown as the mean±S.D. (*n*=2). (**b**) Picture of SCID mice with tumors at 4 weeks after WT ESCs, *Smad3−/−* ESCs and Rif1 shRNA-transduced *Smad3−/−* ESCs were subcutaneously injected into SCID mice. (**c**) Genotyping of ES cell formed tumors to confirm *Rif1* shRNA integration in *Rif1* shRNA transduced *Smad3*−/− ES cells. (**d**) Quantitative real-time PCR to examine the mRNA levels of *Smad3*, *Rif1*, *Ccnd2*, *Mmp2* and *Mmp9* in tumors grown from WT ESCs, *Smad3−/−* ESCs and Rif1 shRNA-transduced *Smad3−/−* ESCs. *Actin* was analyzed as an internal control. The data are shown as the mean±S.D. (*n*=3). (**e**) Immunofluorescence staining with anti-PCNA antibody to examine PCNA expression in WT ESC, control *Smad3−/−* ESC and Rif1 shRNA-transduced *Smad3−/−* ESC-formed teratomas. The nuclei were stained with DAPI. Scale bar=200 *μ*m. (**f**) Quantification of the PCNA-positive cell percentage compared with DAPI in WT ESC, control *Smad3−/−* ESC and Rif1 shRNA transduced *Smad3−/−* ESC-formed teratomas. Statistically significant differences, calculated through student's *t*-tests, are indicated (*P<0.05; **P<0.01; ***P<0.001)

*Smad3−/−* ESC-differentiated cells show enhanced cell migration.^12^ To examine whether upregulation of Rif1 contributes to cell migration (Supplementary Figure S6A), we performed wound-healing and transwell assays. After induction of ESC differentiation by withdrawing 2i and leukemia inhibitory factor (LIF), a wound scratch was generated. Three hours after the scratch, there was no obvious difference in the wound gap between control and Rif1 shRNA-transduced *Smad3−/−* ESC-differentiated cells. But at 12 h after the scratch, the wound gap of the control sample was narrower than *Rif1* knockdown sample, suggesting a reduced cell migration capacity caused by *Rif1* knockdown. This difference was more obvious at 24 h (Figure 6a). In addition, a transwell assay also revealed that *Rif1* knockdown *Smad3−/−* ESC-differentiated cells had lower migration capacity than the control (Supplementary Figure S6B). This is consistent with the cell migration markers *Mmp2* and *Mmp9* being significantly downregulated in *Rif1* knockdown *Smad3−/−* ESC-differentiated cells compared with the control knockdown samples (Supplementary Figure S6C).

### Rif1 shRNA-transduced *Smad3−/−* ESCs form less-malignant teratomas

To investigate whether upregulation of *Rif1* is responsible for *Smad3−/−* ESCs forming malignant teratoma, we subcutaneously injected WT, *Smad3−/−* ESCs and Rif1 shRNA-transduced *Smad3−/−* ESCs into SCID mice. Four weeks later, all the injected ESCs had formed tumors. *Smad3−/−* ESCs formed the largest tumors and Rif1 shRNA-transduced *Smad3−/−* ESCs formed the smallest tumors (Figure 6b). The same result was obtained by a repeat injection assay. Hematoxylin and eosin staining revealed that all these tumors were teratomas with tissues of three germ layers (Supplementary Figure S6D). Genotyping of tumor samples confirmed the integration of Rif1 shRNA in the teratoma cells produced by Rif1 shRNA-transduced *Smad3−/−* ESCs (Figure 6c). Furthermore, the cell proliferation gene *Ccnd2* and cell migration genes *Mmp2* and *Mmp9* were significantly reduced in the teratomas formed by Rif1 shRNA-transduced *Smad3−/−* ESCs compared with the teratoma formed by control *Smad3−/−* ESCs (Figure 6d). Staining the teratomas with proliferating cell nuclear antigen (PCNA) antibody showed that *Smad3−/−* ESC-formed teratomas contained more PCNA-positive cells than WT ESC and Rif1 shRNA-transduced *Smad3−/−* ESC-formed teratomas (Figures 6e and f). Collectively, these results demonstrated that upregulation of *Rif1* is one of the main factors in *Smad3−/−* ESC transformation.

## Discussion

**Figure 7:**
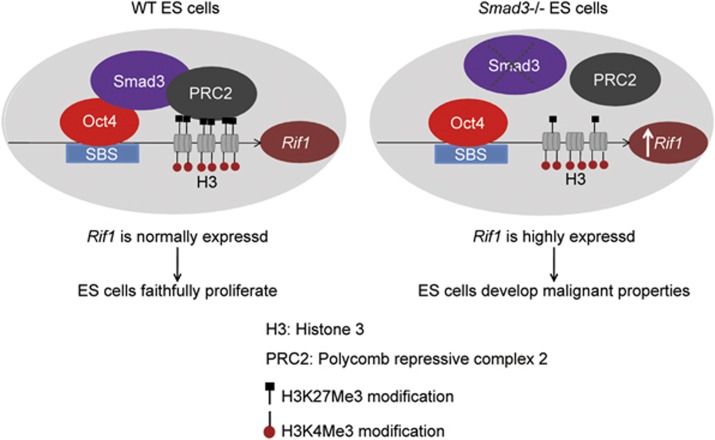
Model for Rif1 regulation and function in ESCs

In this study, we found that Rif1, a factor involved in genomic stability, is tightly regulated by Oct4 and Smad3 in mouse ESCs. Oct4 recruits Smad3 to the *Rif1* promoter and facilitates the loading of the PRC2. To maintain *Rif1* expression at a proper level in ESCs, Oct4 activates *Rif1* expression, but Smad3 is involved in repressing it (Figure 7). Mullen *et al.* reported that Nanog, Oct4 and Sox2 form a complex and tend to co-bind with Smad3 on many genes. From the ChIP-seq data of their report, we found that Nanog, Oct4 and Sox2 all bind to the SBE of *Rif1.*^4^ It is therefore likely that *Rif1* is synergistically regulated by these core transcription factors in ESCs. From past reports and our own studies, it seems very important to keep *Rif1* expressed at a suitable level to sustain ESC pluripotency and stability. Low level of *Rif1* leads to mESC differentiation.^35^ However, high level of *Rif1* is also deleterious to ESCs by driving malignant transformation of *Smad3−/−* ESCs. Comparison of the expression of *Smad3* and *Rif1* between teratoma cells and teratocarcinoma cell lines F9 and P19 revealed that *Smad3* was significantly lower in teratocarcinoma cells than teratoma cells, whereas *Rif1* is significantly higher in teratocarcinoma cells than teratoma cells (Supplementary Figures S7A and S7B). These data support our findings and indicate that a disturbance in the expression of *Smad3* and *Rif1* may be one of the underlying mechanisms for teratocarcinoma formation.

It is reported that Rif1 colocalizes with DNA double-strand breaks (DSBs) and is involved in DNA repair.^48^ Recent studies revealed that Rif1 contributes to the inhibition of 5′ end resection of DSBs, the first step of homologous recombination (HR). As a result, NHEJ, an error-prone DNA repair is promoted with Rif1 presence. In the absence of Rif1, the level of misjoined chromosomes is significantly reduced.^20, 21, 23, 49^ NHEJ leads to more chromatin instabilities, such as deletions, translocations and amplifications, than HR. Chromatin instability is closely linked to cancer cell formation as it enables rapid evolution of cell subclones that show enhanced proliferation, migration and resistance to drug treatment.^50^ Owing to the important role of Rif1 in NHEJ, it is not surprising that it acts as an anti-apoptosis factor and is linked with tumor formation.^51, 52, 53^ The Rif1 level is found to be significantly increased in the breast cancer cells and depletion of Rif1 makes them more sensitive to drug treatment.^51^ Here we observed that Rif1 is highly upregulated in *Smad3−/−* ESCs, which also adopt some cancer cell-like properties. Upregulation of Rif1 leads to enhanced DNA repair, most likely through NHEJ. Therefore, the chromatin of *Smad3−/−* ESCs may be more unstable than WT ESCs and knockdown of *Rif1* in *Smad3−/−* ESC may just slow down the pace of the cells from evolving into more malignant cells. It has been found that *Rif1* depletion can sensitize cancer cells to drug treatment with enhanced apoptosis.^51^ Recent study revealed that Rif1 is important in maintaining the telomere stability in ESCs as it can repress Zscan4, which can trigger hyper-telomere elongation and cell senescence from an elevated expression.^18^ Interestingly, the Rif1 shRNA-transduced *Smad3−/−* ESCs formed smaller teratomas than WT and *Smad3−/−* ESCs. This might be because the constant expression of shRNA of *Rif1* triggers apoptosis and cell senescence. On the basis of these results, it is worthwhile investigating whether control of Rif1 levels by drugs could benefit the treatment of teratocarcinoma.

## Materials and methods

### Cell culture and differentiation

Derivation of WT and *Smad3−/−* mouse ESCs was described in previous reports.^12, 54^ The ESCs were maintained on feeders under the normal ESC medium, which is composed of DMEM with high glucose (Gibco, Life Technologies, Grand Island, NY, USA) supplemented with 15% ES culture grade FBS (Gibco), 0.1 mM non-essential amino acids (NEAA; Gibco), 0.1 mM 2-mercaptoethanol (Gibco), 2 mM glutamine (Gibco), 100 U/ml penicillin and streptomycin (Gibco) and 1000 U/ml LIF (Millipore, Billerica, MA, USA). To obtain feeder-free ESC lines, ESCs were passaged 2–3 times to completely get rid of the feeder cells and grown on a 0.2% gelatin (Sigma, St. Louis, MO, USA)-coated dish in ES medium containing LIF and 2i (1 *μ*M PD325901 and 3 *μ*M CHIR99021).

For ESC differentiation assay, 2i and LIF were removed and 1 μM retinoic acid (RA) was added in the culture medium to induce ESC differentiation. For EB formation assay, monolayer undifferentiated WT ESCs and *Smad3−/−* ESCs were trypsinized into single cells, and then seeded, at a density of 1 × 10^6^ cells/10 ml, in a 10 cm non-adherent dish in ESC culture medium devoid of 2i and LIF. The culture medium was changed every 2 days and the EBs collected on days 2, 4, 6, 8 and 10.

### Plasmid constructs

Two shRNA constructs targeting *Rif1* were generated according to previous reports with pSuper.puro vector (Addgene, Cambridge, MA, USA).^35^ To generate lentiviral vector for express shRNA by lentivirus, Rif1 shRNA sequences together with the H1 promoter were cut from pSuper plasmid with EcoRI and ClaI and sub-cloned to pLVTH plasmid. To construct the Rif1 promoter reporter plasmid, a 2000 bp fragment, encompassing the Smad3-binding sites, was amplified by primers from the genomic DNA of mouse ESCs and cloned into a pGL3 vector (Promega, Madison, WI, USA) at the *Mlu*I and *Xho*I sites. To construct the pCAG-*GFP*, pCAG-*Smad3* and pCAG-*Pou5f1* plasmids, the ORF sequences of these genes were amplified from the cDNA of mouse ESCs, digested and inserted into pCAG-Flag vector (Addgene) at *Bgl*II-*Xho*I (*GFP* and *Smad3*) and *Mlu*I-*Xho*I (*Pou5f1*) sites. All the amplification primers have been added to Table 1 and these recombinant vectors have been sequenced.

### Real-time PCR assay

Real-time PCR analysis was conducted using the ABI Prism 7900HT (Applied Biosystems, Foster City, CA, USA) analysis machine with SYBR Premix Ex Taq (TaKaRa, Shiga, Japan) according to the manufacturer's instructions. The cycle (*C*_T_) values of target genes were first normalized against the *C*_T_ value of an internal control (*Actin* gene) and then normalized against the *C*_T_ value of corresponding transcripts of the control sample. The DNA primer sequences used for the real-time PCR assay are listed in Table 1. For each pair of the primer, only one correct size band and one peak were detected. All the real-time PCR assays comprised triplicate data with samples from three independent experiments.

### Western blot

WT and *Smad3−/−* ESCs were collected with RIPA protein lysis buffer, containing 0.2 M NaCl, 1% SDS, 1 mM PMSF inhibitor and 0.1 M DTT. Then, the SDS-PAGE was used to separate the proteins. After separation, the proteins were transferred to PVDF membranes (Pall Corporation, Port Washington, NY, USA). Subsequently, the PVDF membranes were blocked in 5% nonfat milk (BD Company, Franklin Lakes, NJ, USA) in TBS with 0.1% Tween-20 for 1 h at room temperature and incubated with the primary antibody in TBS+0.1% Tween-20 overnight at 4 °C. The primary antibodies and the dilution ratios were as follows: Mouse anti-Gapdh (A-3) (sc-137179; Santa Cruz Biotechnology, Santa Cruz, CA, USA) used at 1/2000; Rabbit anti-Smad3 antibody (06–920, Upstate, Billerica, MA, USA) used at 1/1000; Goat anti-Oct3/4 (N-19) (sc-8628; Santa Cruz Biotechnology) used at 1/2000; Rif1 antibody (provided by Ian R Adams) used at 1/1000 dilution; Anti-Replication Protein A (Ab-2) (RPA34-19) antibody (Millipore) used at a concentration of 5 μg/ml; and Rabbit anti-pH2AX (Ser139, #2577), anti-pChk1 (Ser345, #2348) and anti-pChk2 (Thr68, #2661; Cell Signaling Technology, Denver, MA, USA) used at 1/1000 dilution. After blotting with the primary antibody, the PVDF membranes were washed with TBS+0.1% Tween 205 times, for 10 min each, and then blotted with the proper secondary antibodies. The secondary antibodies were anti-mouse immunoglobulin G (IgG)-HRP (Sant Cruz Biotechnology; Sc-2314) and anti-Rabbit IgG-HRP (GE Healthcare Life Sciences Dako, Piscataway, NJ, USA) at 1/10000 dilutions for both. Eventually, the signals were tested using ECL detection reagents (Abcam, Cambridge, UK).

### Microarray

Total RNAs of WT ESCs and *Smad3−/−* cells were extracted using Trizol (Invitrogen, Life Technologies) according to the manufacturer's instructions. Ten  μg of total RNAs was aliquoted and digested with DNAse (New England Biolaboratory, Ipswich, MA, USA) at 37 °C for 45 min to remove DNA contamination, and then the RNA samples were purified with a RNeasy mini-kit (Qiagen, Valencia, CA, USA) according to the manufacturer's instructions. Subsequently, the purified RNAs were transferred for reverse transcription, labeled and hybridized to an Affymetrix mouse exon 1.0 ST Array (Santa Clara, CA, USA) according to the manufacturer's instructions. The array data were analyzed using Partek Inc (St. Louis, MO, USA) and Genespring software (Agilent Technologies Inc, Santa Clara, CA, USA). The threshold for gene expression was 1.5-fold. The expression level of genes larger than 1.5-fold (up or down) were picked out to draw the heat map. The GEO access number for the microarray data is GSE57995.

### ChIP assay

A ChIP assay for mouse ESCs was carried out as described previously.^55^ Briefly, 1% (w/v) formaldehyde was added to 3 × 10^7^ cells and incubated at room temperature for 10 min, then inactivated by adding 125 mM glycine for 5 min. The cells were then lysed and the chromatin fragmented by a Bioruptor Sonicator (Bioruptor UCD-200, Diagenode Company, Liège, Belgium) to a size around 500 bp. Soluble chromatins were incubated at 4 °C overnight with a Dynabead (Invitrogen) coupled anti-Smad3 antibody (06–920, Upstate), anti-Oct3/4 (N-19) (sc-8628; Santa Cruz Biotechnology), anti-H3K9Me2 antibody (Milipore), anti-H3K9Me3 antibody (Milipore), anti-H3K4Me3 antibody (Abcam), anti-H3K27Me3 antibody (Milipore), anti-SUZ12 antibody (Abcam) or a corresponding control IgG (Milipore). The antibody-enriched DNAs were decrosslinked and purified with phenol–chloroform (Ambion, Applied Biosystems), followed by ethanol precipitation. The precipitated DNA was dissolved in TE buffer and analyzed by real-time PCR using the ABI Prism 7900HT sequence detection system and SYBR Premix Ex Taq (TaKaRa). Fold enrichments of the enriched DNA were calculated according to ratios of the immunoprecipitated DNA to the input samples and then normalized against the DNA level at control regions. All the DNA primer sequences used for the ChIP-qPCR assay are listed in Table 1. For each pair of the primer, only one correct size band and one peak were detected. All the real-time PCR assays comprised triplicate data with samples from three independent experiments.

### Luciferase assay

For luciferase assay, the Renilla plasmid (5 ng per well) was used as the internal transfection control, whereas the pGL3 empty vector (100 ng per well) was used as the experimental control. Lipfectamine 2000 (Invitrogen) was used to conduct transfection experiments following the manufacturer's instructions. Forty-eight hours after transfection, luciferase activity was detected using a Dual-Luciferase Reporter Assay System (Promega) according to the manufacturer's instructions. Transfection of each pGL3 construct was performed in triplicate in each assay and a total of three independent experiments were performed. Empty vector (pGL3) was transfected to both WT and *Smad3−/−* ESCs in triplicate for normalization. The luciferase readings were recorded and ratios of Renilla luciferase readings to firefly luciferase readings were recorded for each experiment and triplicate data averaged. The average values of the tested constructs were normalized to the activity of the pGL3 empty construct and the Renilla activity. The error bar represents mean±S.D. (*n*=3).

### AP stain

WT and *Smad3−/−* ESCs were washed with DPBS buffer (Dulbecco's phosphate-buffered saline, Invitrogen) and fixed with a solution containing 90% methanol and 10% formaldehyde for 20 min at room temperature. The ESC samples were then washed with DPBS buffer three times, each for 3 min. Subsequently, the ESCs were incubated for 15 min in the dark chamber at room temperature with an AP staining solution composed of solution A (0.4 mg/ml Fast Red Violet LB Base solution, Sigma), solution B (4 mg/ml Napthol AS-BI phosphate solution, Sigma) and water, in a ratio of 2 : 1 : 3, according to the manufacturer's instructions. Finally, the ESC samples were washed with DPBS buffer three times, each for 3 min, and photographed with an Olympus microscope (FV1000, Olympus, Tokyo, Japan).

### BrdU integration and flow cytometry analysis

3 × 10^5^ WT ESCs and *Smad3−/−* ESCs, respectively, were seeded on six-well plates in an ESC culture medium for 2 days and then incubated in a serum-free ESC culture medium for one night. The next morning, the medium was replaced with a thin layer of PBS buffer and the cells were irradiated with UV (40 mJ cm^−2^) using UV Stratalinker 2400 (Stratagene Company, Oceanside, CA, USA). After UV irradiation, the cells were cultured in normal conditions (37 °C and 5% CO_2_) for another 3 h in ESC culture medium containing serum. Thirty minutes before collecting the samples, then BrdU (10 *μ*M) was added in the medium for pulse integration. The WT ESCs and *Smad3−/−* ESCs were then digested with 0.25% EDTA-trypsin to obtain single cells, which were subsequently fixed in cold 70% ethanol, overnight at 4 °C. To remove RNA contamination, WT and *Smad3−/−* ESC samples were treated with 100 *μ*g/ml RNAse (Sigma) for 30 min at 37 °C. Next, the cells were stained with the Alexa Fluor 488 conjugate anti-BrdU mouse monoclonal antibody (1 : 100, Invitrogen) for 1 h at room temperature. Eventually, the cells were washed with PBS and stained with 20 *μ*g/ml PI solution (Sigma) at 37 °C for 1 h in a dark room. Flow cytometric analysis was performed on 10,000 gated events using a FACS Calubur (BD Biosciences, Becton Dickinson, Franklin Lakes, NJ, USA). The software used to analyze the phase distribution of the cell cycle was FCS Express V3 (De Novo Software Company, Los Angeles, CA, USA). The cell distribution ratio data were collected and analyzed. All the data were duplicated.

### Wound-healing assay and transwell assay

4 × 10^5^ WT and *Smad3−/−* ESCs were seeded in six-well tissue culture plates, respectively, in 2i and LIF ESC culture media. After 2 days, the ESC media were changed to differentiation media, in which 2i and LIF were removed and 1 *μ*M RA (Sigma) was added, for another day to promote quick ESC differentiation. When ESCs were completely differentiated into monolayer cells, autoclaved yellow pipette tips were used to generate scratches. After scratching, the detached cells were removed by washing twice with DPBS buffer, and then cultured in differentiation media for another 24 h under normal condition. Images were taken by a Zeiss microscope (Carl Zeiss, Jena, Germany) and analyzed, using Image J software (NIH, Washington, DC, USA), at 0 and 24 h. Triplicate independent assays were performed. The cell invasive assay was performed using the CytoSelect 24-Well Cell Invasion Assay Kit (Cat # CBA-110-COL, Cell Biolabs, Inc., San Diego, CA, USA) according to the manufacturer's instructions.

## Figures and Tables

**Table 1:** Primers used in the study

*Real-time PCR primers*	
Actin F	5′-ACCAACTGGGACGACATGGAGA-3′
Actin R	5′-TACGACCAGAGGCATACAGGGAC-3′
Smad3 F	5′-CTGGGCCTACTGTCCAATGT-3′
Smad3 R	5′-CATCTGGGTGAGGACCTTGT-3′
Oct4 F	5′-AAGCCTGCCAGGAGCAAA-3′
Oct4 R	5′-ATCCGGCGTTATGCTGCTCT-3′
Nanog F	5′-GGCTATCTGGTGAACGCATCTGGAAG-3′
Nanog R	5′-AACTGTACGTAAGGCTGCAGAAAGTCCTC-3′
Rif1 F	5′-ACTGTCTCCACGGATGAAGA-3′
Rif1 R	5′-CAAATAGCTGGCTTCCAGTG-3′
Lefty1 F	5′-TGTGTGTGCTCTTTGCTTCC-3′
Lefty1 R	5′-GGGGATTCTGTCCTTGGTTT-3′
Lefty2 F	5′-CAGCCAGAATTTTCGAGAGGT-3′
Lefty2 R	5′-CAGTGCGATTGGAGCCATC-3′
Ccnd2 F	5′-AAGCCTGCCAGGAGCAAA-3′
Ccnd2 R	5′-ATCCGGCGTTATGCTGCTCT-3′
Cdx2 F	5′-CCTGCGACAAGGGCTTGTTTAG-3′
Cdx2 R	5′-TCCCGACTTCCCTTCACCATAC-3′
Pax6 F	5′-GCATGCAGAACAGTCACAGCGGA-3′
Pax6 R	5′-ACTCCCGTTTATACTGGGCTATTT-3′
Hand1 F	5′-GCCAAGGATGCACAAGCA-3′
Hand1 R	5′-GGGCTGCTGAGGCAACTC-3′
Fgf5	5′-GAGAGTGGTACGTGGCCCTGAACAAGAGAG-3′
Fgf5	5′-CTTCAGTCTGTACTTCACTGGGCTGGGACT-3′
T F	5′-CATCGGAACAGCTCTCCAACCTAT-3′
T R	5′-GTGGGCTGGCGTTATGACTCA-3′
Gata6 F	5′-TGCAAGATTGCATCATGACAGA-3′
Gata6 R	5′-TGACCTCAGATCAGCCACGTTA-3′
Sox17 F	5′-TTCTGTACACTTTAATGAGGCTGTTC-3′
Sox17 R	5′-TTGTGGGAAGTGGGATCAAG-3′
Cxcr4 F	5′-AGCATGACGGACAAGTACC-3′
Cxcr4 R	5′-GATGATATGGA AGCCTTACAC-3′
Mmp2 F	5′-ATGATGACATCAAGGGGATC-3′
Mmp2 R	5′-CGCCAAATAAACCGGTCCTT-3′
Mmp9 F	5′-GAGCTGTGCGTCTTCCCCTTC-3′
Mmp9 R	5′-GGAATGATCTAAGCCCAGTGC-3′
Eomes F	5′-CCTGGTGGTGTTTTGTTGTG-3′
Eomes R	5′-TTTAATAGCACCGGGCACTC-3′
Elf5 F	5′-CCCTCCTCCTCTTCAAAACC-3′
Elf5 R	5′-AAGTTGCCACAAGACCATCC-3′
Pdgfra F	5′-ACGTTCAAGACCAGCGAGTT-3′
Pdgfra R	5′-CGATCGTTTCTCCTGC CTTA-3′
CGA F	5′-GCCAGAGTGGAGAATCATAC-3′
CGA R	5′-AACTGAAGCGCGTCAGAAGT-3′
	
*ChIP-qPCR primers*	
Actin F	5′-GTTACCCGGGATACTGACCT-3′
Actin R	5′-GGCACCACACCTTCTAC-3′
Lefty1 F	5′-GTAGCCAGCAGACAGGACAA-3′
Lefty1 R	5′-ATCCCCAATCCACATTCA-3′
Lefty2 F	5′-GCAATCTGCCCACTGTAAAA-3′
Lefty2 R	5′-TCGATCTTCCCAAGACTC-3′
Rif1-1 F	5′-CCAATTCTAGGCAGTTGCCT-3′
Rif1-1 R	5′-GGGAGTGTTGCTAAAGG-3′
Rif1-2 F	5′-ATCTCTGTGTTTGAGCACCC-3′
Rif1-2 R	5′-CGTGGAATCTTTCCGTCC-3′
	
*shRNA sequences*	
Rif1 shRNA1 F	5′-GATCCCCGAACCGTATTCAGAATCAAttcaagagaTTGATTCTGAATACGGTTCTTTTTA-3′
Rif1 shRNA1 R	5′-AGCTTAAAAAGAACCGTATTCAGAATCAAtctcttgaaTTGATTCTGAATACGGTTCGGG-3′
Rif1 shRNA2 F	5′-GATCCCCGAGTACAATAAGTGTTGATttcaagagaATCAACACTTATTGTACTCTTTTTA-3′
Rif1 shRNA2 R	5′-AGCTTAAAAAGAGTACAATAAGTGTTGATTctcttgaaaTCAACACTTATTGTACTCGGG-3′
	
*Rif1 promoter primers*	
Rif1 F	5′-GTGGTCACGCGTTGTAGTTCTGAGTCTCTGG-3′
Rif1 R	5′-ACGTCACTCGAGGCTAGAGATGGGTGATGTA-3′
	
*cDNA clone primers*	
GFP F	5′-ATACCGAGATCTATGGTGAGCAAGGGCGAGGAG-3′
GFP R	5′-ATACCCCTCGAGCTATCGAGATCTGAGTCCGGAC-3′
Smad3 F	5′-GTGGTCAGATCTATGTCGTCCATCCTGCCCT-3′
Smad3 R	5′-ACGTCACTCGAGCTAAGACACACTGGAACAGC-3′
Pou5f1 F	5′-GTGGTCACGCGTATGGCTGGACACCTGGCTT-3′
Pou5f1 R	5′-ACGTCACTCGAGTCAGTTTGAATGCATGGGAG-3′
	
*Genotyping primers*	
pLvth-Rif1 shRNA F	5′-CGCTGACGTCATCAACCCGCTCCAAGGA-3′
pLvth-Rif1 shRNA R	5′-CGTATAATGTATGCTATACGAAG-3′

## Abbreviations

AP: alkaline phosphatase
ATM: ataxia telangiectasia mutated
ATR: ataxia telangiectasia mutated and Rad3-related protein
BrdU: bromodeoxyuridine
Ccnd2: cyclin-D2
ChIP: chromatin immunoprecipitation
EB: embryoid body
ESC: embryonic stem cell
HR: homologous recombination
IgG: immunoglobulin G
LIF: leukemia inhibitory factor
MEF: mouse embryonic fibroblast
NHEJ: non-homologous end joining
PCNA: proliferating cell nuclear antigen
PI: propidium iodide
RA: retinoic acid
Rif1: RAP1-associated protein 1
RPA: replication protein A
shRNA: short hairpin RNA
Smad3: SMAD family member 3
TGF-*β*: transforming growth factor beta
UV: ultraviolet
WT: wild type
