# Supplementary material for: A tight control of Rif1 by Oct4 and Smad3 is critical for mouse embryonic stem cell stability
Source: Cell Death Dis. 2015 Jan 8;6(1):e1588–. doi: 10.1038/cddis.2014.551 (PMC4669749; doi:10.1038/cddis.2014.551)
Supplement: Supplementary Information [file cddis2014551x1.pdf]

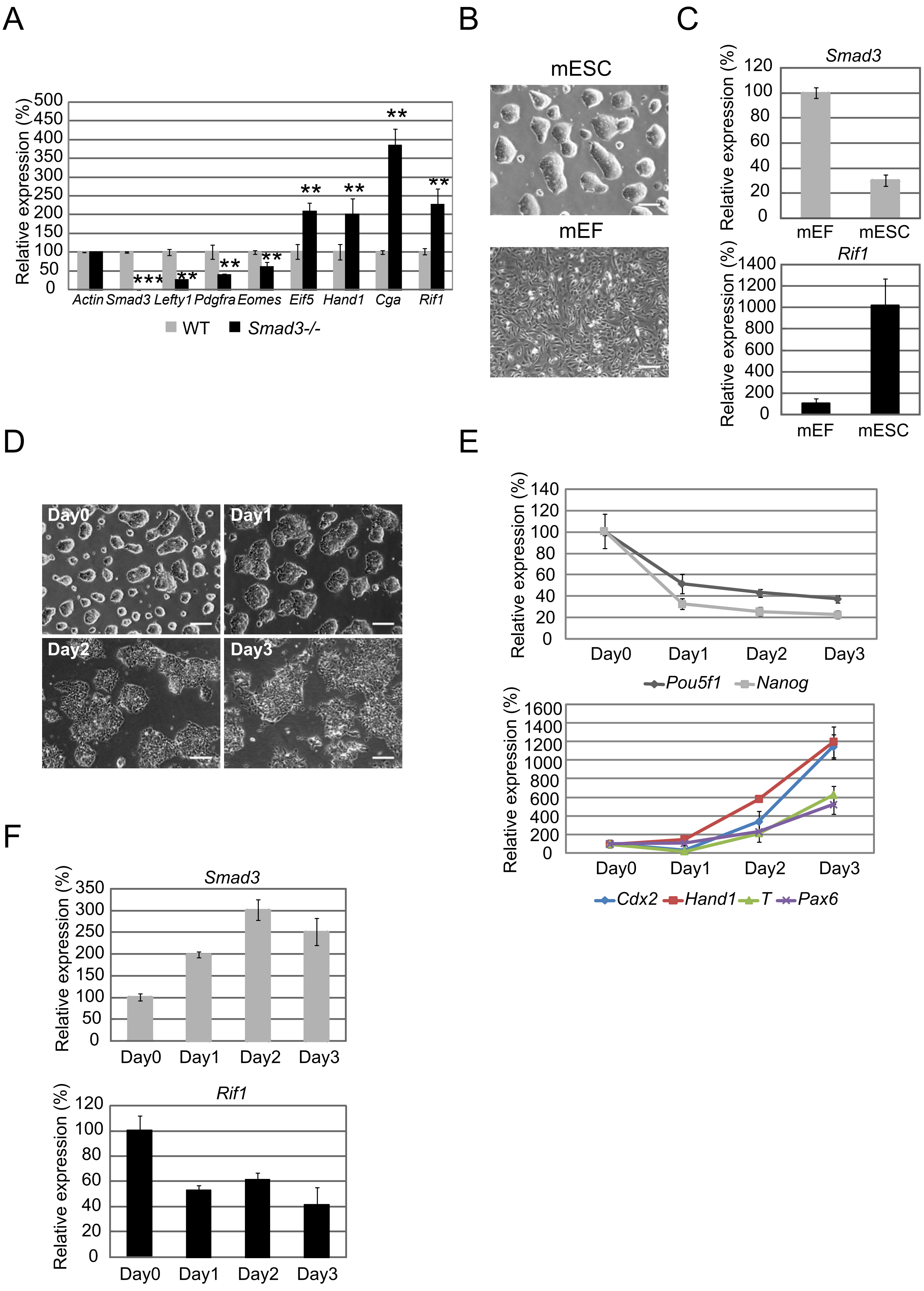
A
Relative expression (%)
500
450
400
350
300
250
200
150
100
50
0
***
**
**
**
**
**
**
**
Actin
Smad3
Lefty1
Pdgfra
Eomes
Eif5
Hand1
Cga
Rif1
WT
Smad3-/-
B
mESC
mEF
C
Smad3
Relative expression (%)
120
100
80
60
40
20
0
mEF
mESC
Rif1
Relative expression (%)
1400
1200
1000
800
600
400
200
0
mEF
mESC
D
Day0
Day1
Day2
Day3
E
Relative expression (%)
140
120
100
80
60
40
20
0
Day0
Day1
Day2
Day3
Pou5f1
Nanog
Relative expression (%)
1600
1400
1200
1000
800
600
400
200
0
Day0
Day1
Day2
Day3
Cdx2
Hand1
T
Pax6
F
Smad3
Relative expression (%)
350
300
250
200
150
100
50
0
Day0
Day1
Day2
Day3
Rif1
Relative expression (%)
120
100
80
60
40
20
0
Day0
Day1
Day2
Day3

Figure S1

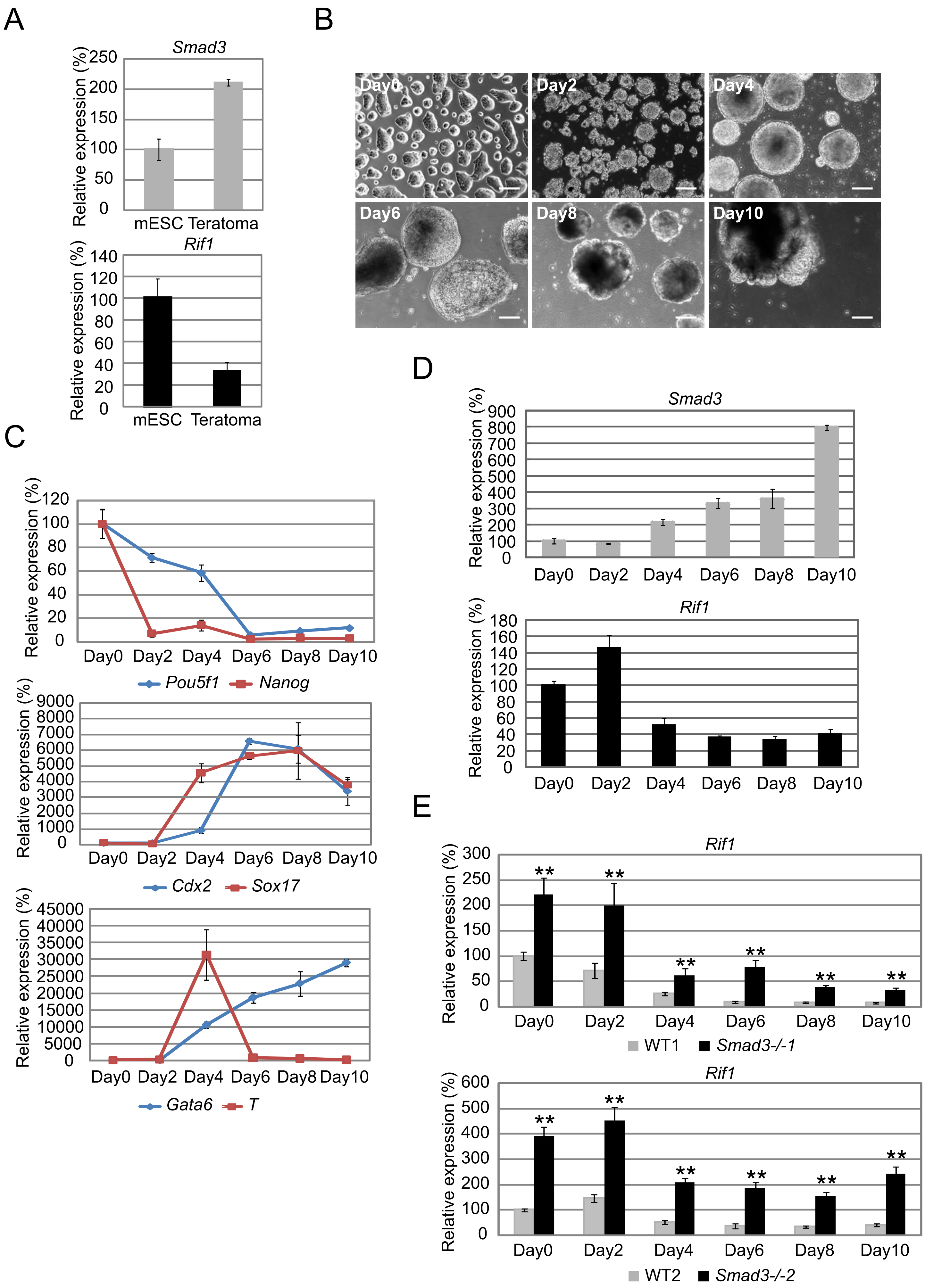
A
Smad3
Relative expression (%)
250
200
150
100
50
0
mESC Teratoma
Rif1
Relative expression (%)
140
120
100
80
60
40
20
0
mESC Teratoma
B
Day0
Day2
Day4
Day6
Day8
Day10
C
Relative expression (%)
120
100
80
60
40
20
0
Day0 Day2 Day4 Day6 Day8 Day10
Pou5f1 Nanog
Relative expression (%)
9000
8000
7000
6000
5000
4000
3000
2000
1000
0
Day0 Day2 Day4 Day6 Day8 Day10
Cdx2 Sox17
Relative expression (%)
45000
40000
35000
30000
25000
20000
15000
10000
5000
0
Day0 Day2 Day4 Day6 Day8 Day10
Gata6 T
D
Smad3
Relative expression (%)
900
800
700
600
500
400
300
200
100
0
Day0 Day2 Day4 Day6 Day8 Day10
Rif1
Relative expression (%)
180
160
140
120
100
80
60
40
20
0
Day0 Day2 Day4 Day6 Day8 Day10
E
Rif1
Relative expression (%)
300
250
200
150
100
50
0
**
Day0 Day2 Day4 Day6 Day8 Day10
WT1 Smad3-/-1
Rif1
Relative expression (%)
600
500
400
300
200
100
0
**
Day0 Day2 Day4 Day6 Day8 Day10
WT2 Smad3-/-2

Figure S2

A

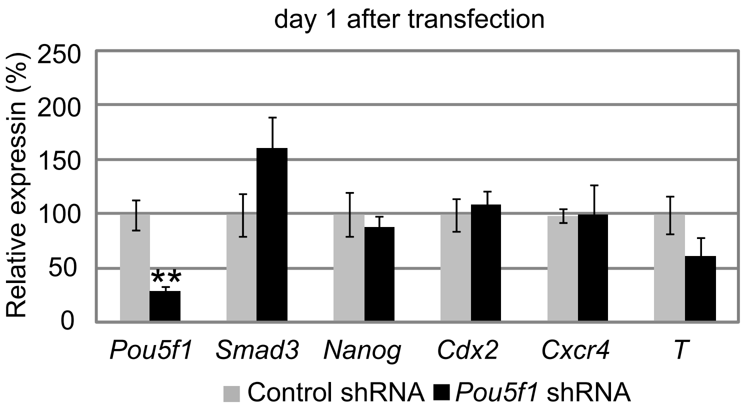
day 1 after transfection
Relative expressin (%)
250
200
150
100
50
0
**
Pou5f1
Smad3
Nanog
Cdx2
Cxcr4
T
Control shRNA
Pou5f1 shRNA

B

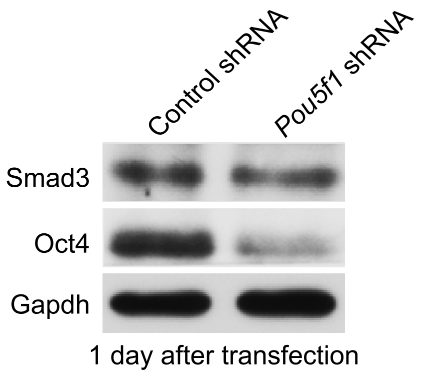
Control shRNA
Pou5f1 shRNA
Smad3
Oct4
Gapdh
1 day after transfection

Figure S3

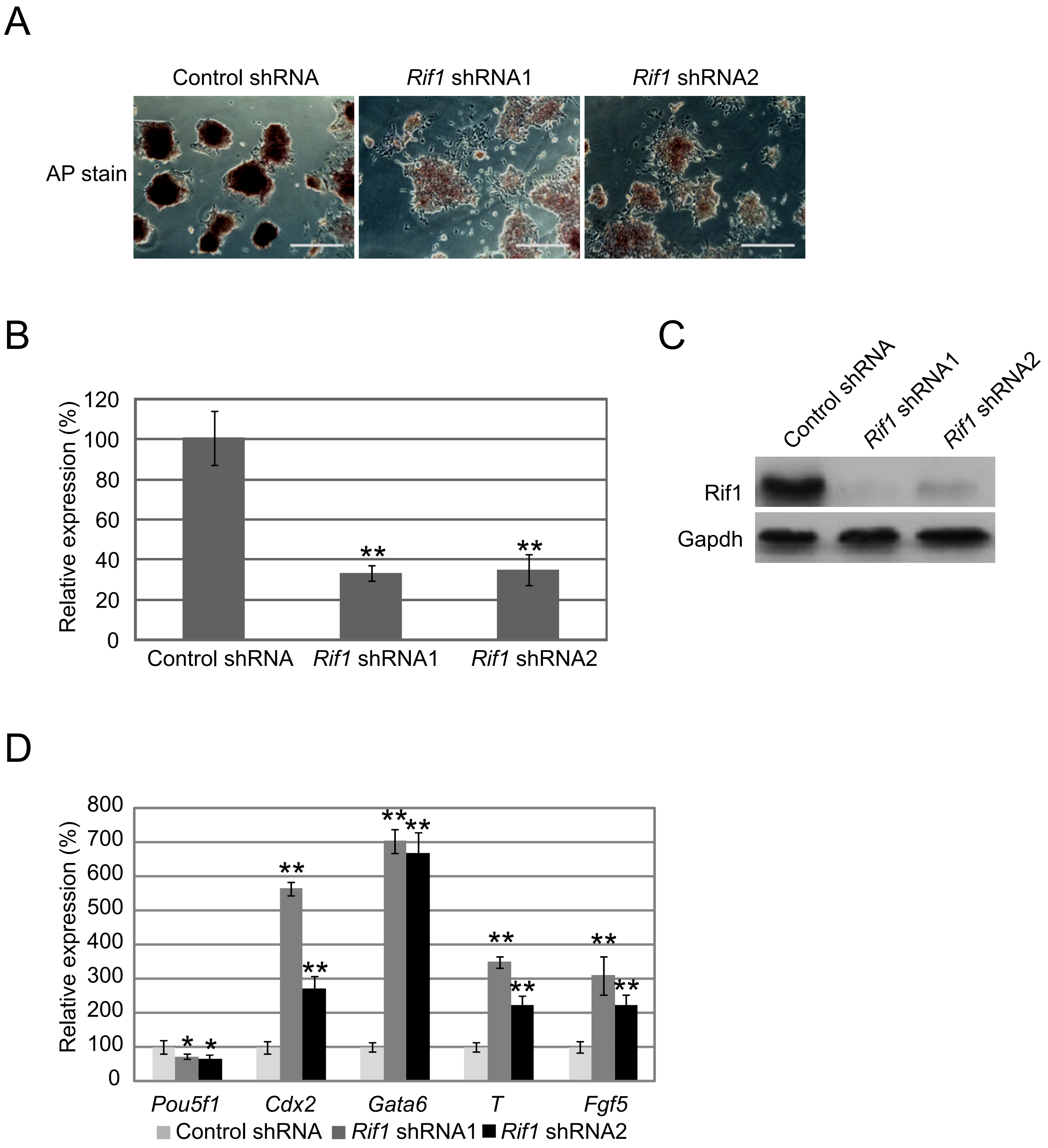
A
Control shRNA
Rif1 shRNA1
Rif1 shRNA2
AP stain
B
Relative expression (%)
120
100
80
60
40
20
0
**
**
Control shRNA
Rif1 shRNA1
Rif1 shRNA2
C
Control shRNA
Rif1 shRNA1
Rif1 shRNA2
Rif1
Gapdh
D
Relative expression (%)
800
700
600
500
400
300
200
100
0
Pou5f1
Cdx2
Gata6
T
Fgf5
Control shRNA
Rif1 shRNA1
Rif1 shRNA2

Figure S4

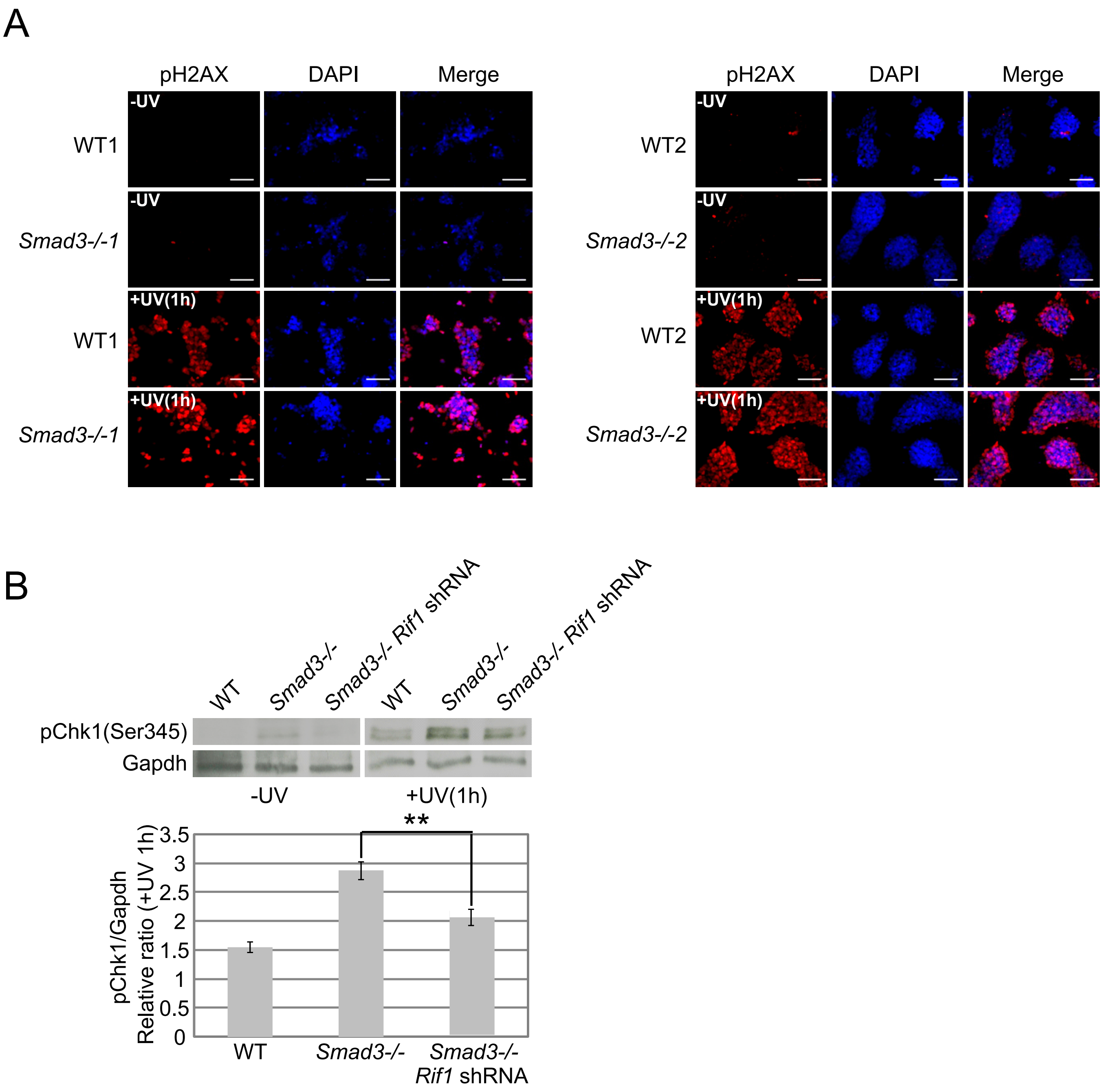
A
pH2AX
DAPI
Merge
-UV
+UV(1h)
WT1
Smad3-/-1
WT2
Smad3-/-2
B
WT
Smad3-/-
Smad3-/- Rif1 shRNA
pChk1(Ser345)
Gapdh
-UV
+UV(1h)
**
pChk1/Gapdh
Relative ratio (+UV 1h)
3.5
3
2.5
2
1.5
1
0.5
0
WT
Smad3-/-
Smad3-/- Rif1 shRNA

Figure S5

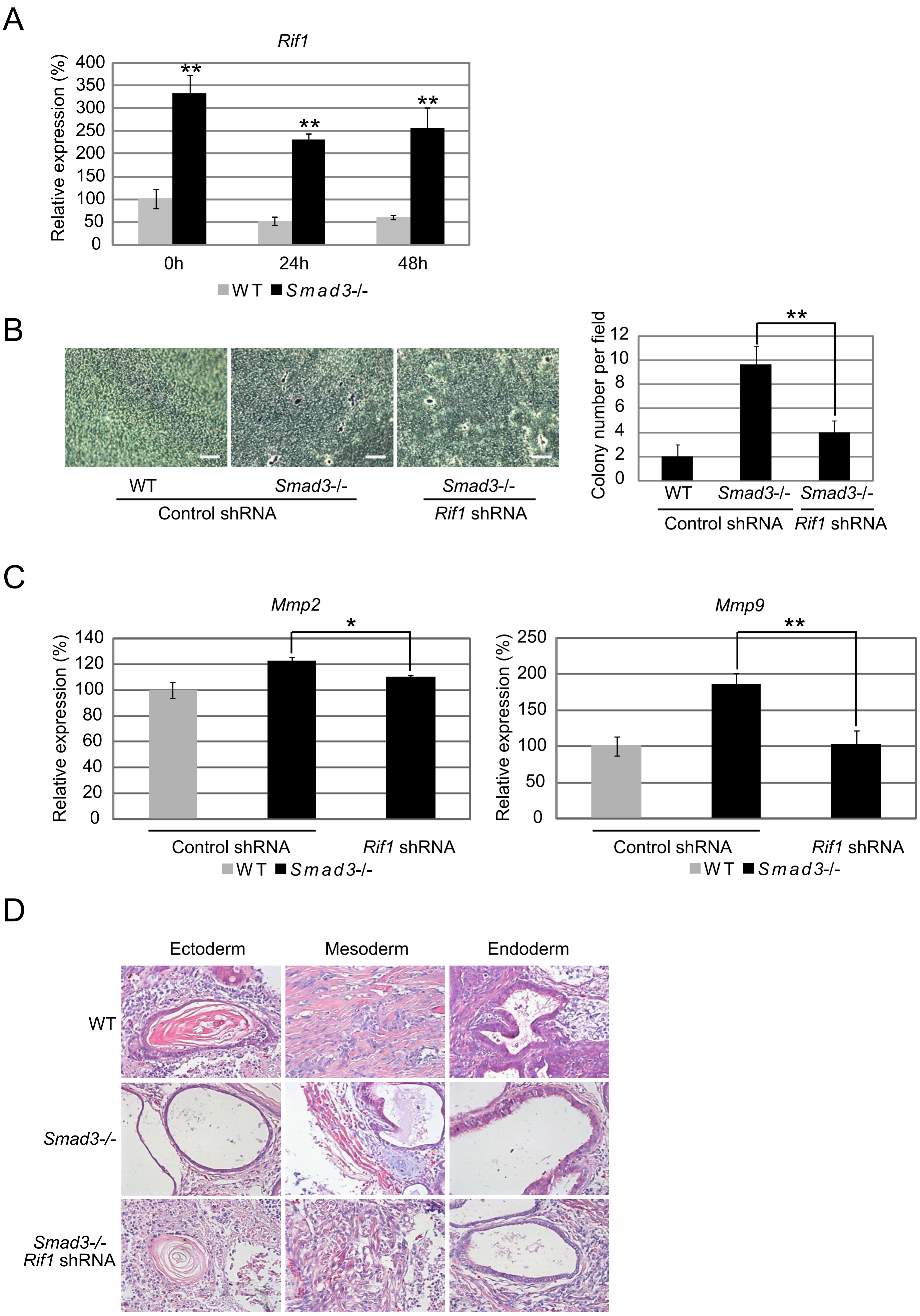
A
Rif1
Relative expression (%)
400
350
300
250
200
150
100
50
0
**
**
**
0h
24h
48h
WT
Smad3-/-
B
WT
Smad3-/-
Control shRNA
Smad3-/-
Rif1 shRNA
Colony number per field
12
10
8
6
4
2
0
**
WT
Smad3-/-
Smad3-/-
Control shRNA
Rif1 shRNA
C
Mmp2
Relative expression (%)
140
120
100
80
60
40
20
0
*
Control shRNA
Rif1 shRNA
WT
Smad3-/-
Mmp9
Relative expression (%)
250
200
150
100
50
0
**
Control shRNA
Rif1 shRNA
WT
Smad3-/-
D
Ectoderm
Mesoderm
Endoderm
WT
Smad3-/-
Smad3-/-
Rif1 shRNA

Figure S6

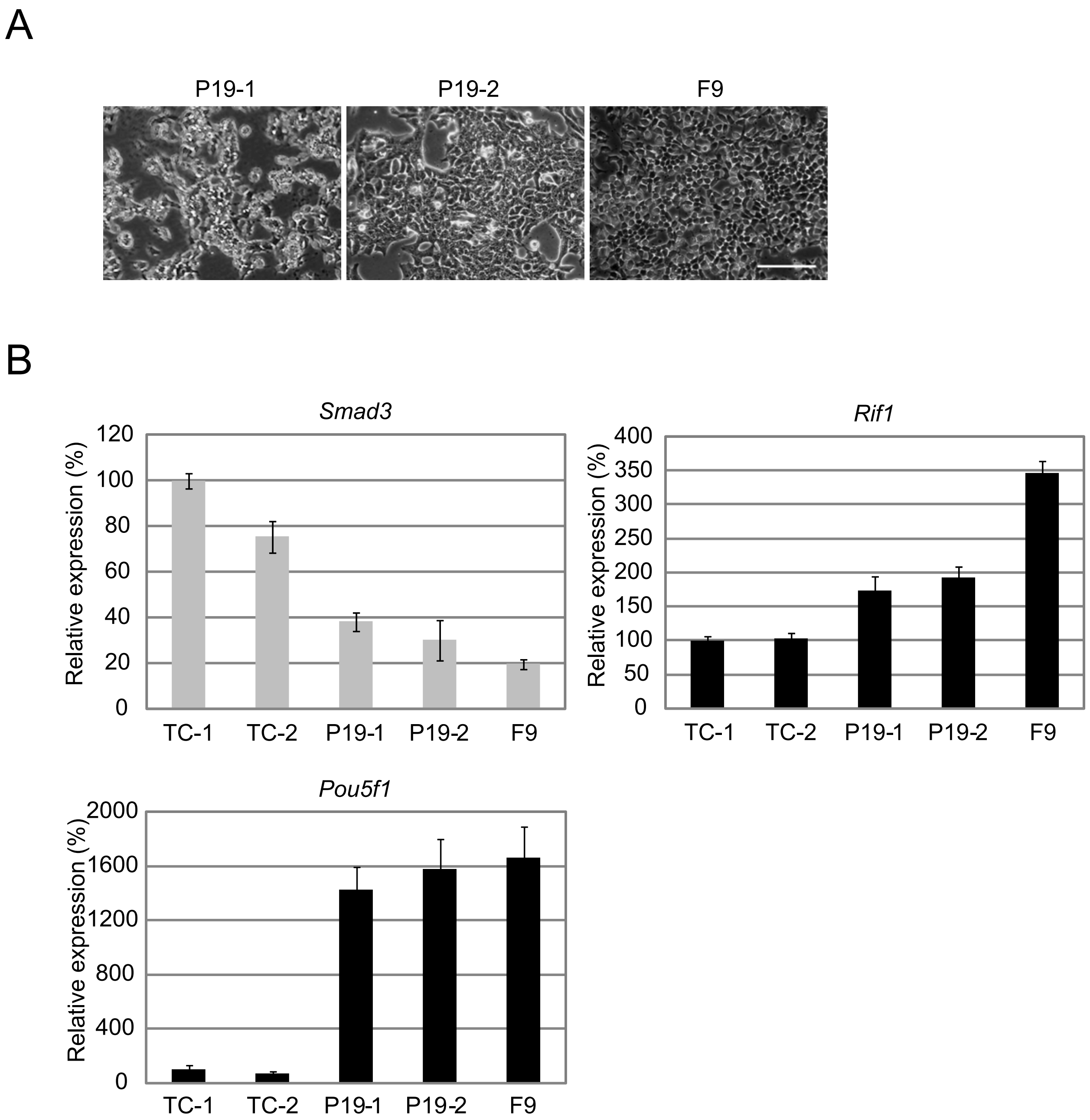
A
P19-1
P19-2
F9
B
Smad3
Relative expression (%)
120
100
80
60
40
20
0
TC-1
TC-2
P19-1
P19-2
F9
Rif1
Relative expression (%)
400
350
300
250
200
150
100
50
0
TC-1
TC-2
P19-1
P19-2
F9
Pou5f1
Relative expression (%)
2000
1600
1200
800
400
0
TC-1
TC-2
P19-1
P19-2
F9

Figure S7

Table S1. Primers used in the study

Real-time PCR primers	
Actin F	5'-ACCAACTGGGACGACATGGAGA-3'
Actin R	5'-TACGACCAGAGGCATACAGGGAC-3'
Smad3 F	5'-CTGGGCCTACTGTCCAATGT-3'
Smad3 R	5'-CATCTGGGTGAGGACCTTGT-3'
Oct4 F	5'-AAGCCTGCCAGGAGCAAA-3'
Oct4 R	5'-ATCCGGCGTTATGCTGCTCT-3'
Nanog F	5'-GGCTATCTGGTGAACGCATCTGGAAG-3'
Nanog R	5'-AACTGTACGTAAGGCTGCAGAAAGTCCTC-3'
Rif1 F	5'-ACTGTCTCCACGGATGAAGA-3'
Rif1 R	5'-CAAATAGCTGGCTTCCAGTG-3'
Lefty1 F	5’-TGTGTGTGCTCTTTGCTTCC-3’
Lefty1 R	5’-GGGGATTCTGTCCTTGGTTT-3’
Lefty2 F	5'-CAGCCAGAATTTTCGAGAGGT-3'
Lefty2 R	5'-CAGTGCGATTGGAGCCATC-3'
Ccnd2 F	5’-AAGCCTGCCAGGAGCAAA-3’
Ccnd2 R	5’-ATCCGGCGTTATGCTGCTCT-3’
Cdx2 F	5'-CCTGCGACAAGGGCTTGTTTAG-3'
Cdx2 R	5'-TCCCGACTTCCCTTCACCATAC-3'
Pax6 F	5'-GCATGCAGAACAGTCACAGCGGA-3'
Pax6 R	5'-ACTCCCGTTTATACTGGGCTATTT-3'
Hand1 F	5'-GCCAAGGATGCACAAGCA-3'
Hand1 R	5'-GGGCTGCTGAGGCAACTC-3'
Fgf5	5'-GAGAGTGGTACGTGGCCCTGAACAAGAGAG-3'
Fgf5	5'-CTTCAGTCTGTACTTCACTGGGCTGGGACT-3'
T F	5'-CATCGGAACAGCTCTCCAACCTAT-3'
T R	5'-GTGGGCTGGCGTTATGACTCA-3'
Gata6 F	5'-TGCAAGATTGCATCATGACAGA-3'
Gata6 R	5'-TGACCTCAGATCAGCCACGTTA-3'
Sox17 F	5'-TTCTGTACACTTTAATGAGGCTGTTC-3'
Sox17 R	5'-TTGTGGGAAGTGGGATCAAG-3'
Cxcr4 F	5’-AGCATGACGGACAAGTACC-3’
Cxcr4 R	5′-GATGATATGGA AGCCTTACAC-3’
Mmp2 F	5’-ATGATGACATCAAGGGGATC-3’
Mmp2 R	5’-CGCCAAATAAACCGGTCCTT-3’
Mmp9 F	5’-GAGCTGTGCGTCTTCCCCTTC-3’
Mmp9 R	5’-GGAATGATCTAAGCCCAGTGC-3’

Eomes F	5'-CCTGGTGGTGTTTTGTTGTG-3'
Eomes R	5'-TTTAATAGCACCGGGCACTC-3'
Elf5 F	5'-CCCTCCTCCTCTTCAAAACC-3'
Elf5 R	5'-AAGTTGCCACAAGACCATCC-3'
Pdgfra F	5’-ACGTTCAAGACCAGCGAGTT-3’
Pdgfra R	5’-CGATCGTTTCTCCTGC CTTA-3’
CGA F	5’-GCCAGAGTGGAGAATCATAC-3’
CGA R	5’-AACTGAAGCGCGTCAGAAGT-3’
**ChIP qPCR primers**	
Actin F	5'-GTTACCCGGGATACTGACCT-3'
Actin R	5'-GGCACCACACCTTCTAC-3'
Lefty1 F	5'-GTAGCCAGCAGACAGGACAA-3'
Lefty1 R	5'-ATCCCCAATCCACATTCA-3'
Lefty2 F	5'-GCAATCTGCCCACTGTAAAA-3'
Lefty2 R	5'-TCGATCTTCCCAAGACTC-3'
Rif1-1 F	5'-CCAATTCTAGGCAGTTGCCT-3'
Rif1-1 R	5'-GGGAGTGTTGCTAAAGG-3'
Rif1-2 F	5'-ATCTCTGTGTTTGAGCACCC-3'
Rif1-2 R	CGTGGAATCTTTCCGTCC
**shRNA sequences**	
Rif1 shRNA1 F	5'-GATCCCCGAACCGTATTCAGAATCAAttcaagagaTTGATTCTGAATACGGTTCTTTTTA-3'
Rif1 shRNA1 R	5'-AGCTTAAAAAGAACCGTATTCAGAATCAAtctcttgaaTTGATTCTGAATACGGTTCGGG-3'
Rif1 shRNA2 F	5'-GATCCCCGAGTACAATAAGTGTTGATttcaagagaATCAACACTTATTGTACTCTTTTTA-3'
Rif1 shRNA2 R	5'-AGCTTAAAAAGAGTACAATAAGTGTTGATtctcttgaaATCAACACTTATTGTACTCGGG-3'
**Rif1 promoter primers**	
Rif1 F	5'-GTGGTCACGCGTTGTAGTTCTGAGTCTCTGG-3'
Rif1 R	5'-ACGTCACTCGAGGCTAGAGATGGGTGATGTA-3'
**cDNA clone primers**	
GFP F	5’-ATACCGAGATCTATGGTGAGCAAGGGCGAGGAG-3’
GFP R	5’-ATACCCCTCGAGCTATCGAGATCTGAGTCCGGAC-3’
Smad3 F	5‘-GTGGTCAGATCTATGTCGTCCATCCTGCCCT-3’
Smad3 R	5‘-ACGTCACTCGAGCTAAGACACACTGGAACAGC-3’
Pou5f1 F	5‘-GTGGTCACGCGTATGGCTGGACACCTGGCTT-3’
Pou5f1 R	5‘-ACGTCACTCGAGTCAGTTTGAATGCATGGGAG-3’
**Genotyping primers**	
pLvth-Rif1 shRNA F	5’-CGCTGACGTCATCAACCCGCTCCAAGGA-3’
pLvth-Rif1 shRNA R	5'-CGTATAATGTATGCTATACGAAG-3'

Supplementary figure legends

Figure S1

(A) Quantitative real-time PCR to validate the mRNA level of 8 genes randomly selected from microarray analysis in WT ES cells and *Smad3-/-* ES cells. *Actin* was analyzed as an internal control. The data are shown as the mean ± S.D (n=3). Statistically significant differences, calculated through student-t tests, are indicated (*, $P<0.05$; **, $P<0.01$; ***, $P<0.001$). The gene expression changes of the selected genes are consistent with the microarray result.

(B) Phase contrast microscopy of mouse ES cells and embryonic fibroblasts. Scale bar equals to 200μm.

(C) mRNA expression level of *Smad3* (blue) and *Rif1* (black) was determined through real-time PCR analysis in mouse ES cells and embryonic fibroblasts (MEF), *Actin* was analyzed as an internal control. The data are shown as the mean ± S.D (n=3).

(D) Phase contrast microscopy of mouse ES cell differentiated cells on withdrawing 2i and LIF from day 0 to day 3. Scale bar equals to 200μm.

(E) mRNA expression levels of pluripotency genes (*Pou5f1* and *Nanog*) and differentiation genes (*Cdx2*, *Hand1*, *T* and *Pax6*) was determined through real-time PCR analysis in mouse ES cells and ES cell differentiated cells under withdrawing 2i and LIF condition from day 0 to day 3. *Actin* was analyzed as an internal control. The data are shown as the mean ± S.D (n=3).

(F) mRNA expression levels of *Smad3* (blue) and *Rif1*(black) were determined through real-time PCR analysis in mouse ES cells and ES cell differentiated cells under withdrawing 2i and LIF condition. *Actin* was analyzed as an internal control. The data are shown as the mean ± S.D (n=3).

Figure S2

(A) mRNA expression levels of *Smad3* (blue) and *Rif1* (black) were determined through real-time PCR analysis in mouse ES cells and teratoma cells. *Actin* was analyzed as an internal control. The data are shown as the mean ± S.D (n=3).

(B) Phase contrast microscopy of mouse embryoid body development from day 0 to day 10. Scale bar equals to 200 μm.

(C) mRNA expression levels of pluripotency genes (*Pou5f1* and *Nanog*) and differentiation genes (*Cdx2*, *T*, *Sox17* and *Gata6*) were determined through real-time PCR analysis in mouse ES cells and mouse embryoid body development from day 0 to day 10. *Actin* was analyzed as an internal control. The data are shown as the mean ± S.D (n=3).

(D) mRNA expression levels of *Smad3* (blue) and *Rif1* (black) were determined through real-time PCR analysis in mouse ES cells and embryoid body cells. *Actin* was analyzed as an internal control. The data are shown as the mean ± S.D (n=3).

(E) Quantitative real-time PCR to examine the mRNA level of *Rif1* in WT ES cell (WT1 and WT2) and *Smad3-/-* (*Smad3-/-1* and *Smad3-/-2*) ES cell formed embryoid body from day 0 to day 10. *Actin* was analyzed as a control. The data are shown as the mean ± S.D (n=3). Statistically significant differences, calculated through student-t tests, are indicated (*, P<0.05; **, P<0.01; ***, P<0.001).

Figure S3

(A) mRNA expression levels of *Pou5f1*, *Nanog*, *Smad3* and differentiation genes (*Cdx2*, *T* and *Cxcr4*) were determined through real-time PCR analysis in mouse ES cells transfected with pSuper shRNA control and pSuper-*Pou5f1*-shRNA plasmids and selected with 1μg/ml puromycin for 1 day. *Actin* was analyzed as an internal control. The data are shown as the mean ± S.D (n=3). Statistically significant differences, calculated through student-t tests, are indicated (*, P<0.05; **, P<0.01; ***, P<0.001).

(B) Western blot analysis of the expression of Oct4 and Smad3 in mouse ES cells transfected with control shRNA and *Pou5f1* shRNA plasmids and selected with 1μg/ml puromycin for 1 day. Gapdh expression level was used as an internal control.

Figure S4

(A) Alkaline phosphatase stain of WT mouse ES cells transfected with pSuper control and pSuper-*Rif1*-shRNA plasmids. Scale bar equals to 200 μm.

(B) Quantitative real-time PCR to examine the mRNA level of *Rif1* in ES cells transfected with pSuper control and pSuper-*Rif1*-shRNA plasmids. *Actin* was analyzed as an internal control. The data are shown as the mean ± S.D (n=3).

Statistically significant differences, calculated through student-t tests, are indicated (*, P<0.05; **, P<0.01; ***, P<0.001).

(C) Western blot analysis of the expression of Rif1 in mouse ES cells transfected with pSuper and pSuper-*Rif1*-shRNA plasmids. Gapdh expression level was used as an internal control.

(D) mRNA levels of the pluripotency gene (*Pou5f1*) and differentiation genes (*Cdx2*, *Gata6*, *T* and *Fgf5*) were determined through real-time PCR analysis in mouse ES cells transfected with pSuper shRNA control and pSuper-*Rif1*-shRNA plasmids and selected with 1μg/ml puromycin for 3 days. *Actin* was analyzed as an internal control. The data are shown as the mean ± S.D (n=3). Statistically significant differences, calculated through student-t tests, are indicated (*, P<0.05; **, P<0.01; ***, P<0.001).

Figure S5

(A) Immunofluorescence stain with pH2AX (Ser139) antibody in WT (WT1 and WT2) and *Smad3-/-* (*Smad3-/-1* and *Smad3-/-2*) ES cells before and after 1 hour recovery for UV (40 mJ/cm^2) irradiation treatment. Scale bar equals to 200μm.

(B) Western blot analysis the expression of pChk1 (Ser345) in WT, *Smad3-/-* and *Rif1* shRNA transduced *Smad3-/-* ES cells before and after 3 hour recovery for UV (40mJ/cm2) irradiation treatment. Gapdh expression level was used as an internal control. Densitometric analysis of the western blot data of pChk1 (Ser345) after UV irradiation treatment. The data are shown as the mean ± S.D (n=2). Statistically significant differences, calculated through student-t tests, are indicated (*, P<0.05; **, P<0.01; ***, P<0.001).

Figure S6

(A) Quantitative real-time PCR to examine the mRNA level of *Rif1* in WT and *Smad3-/-* ES cells under -2i-LIF+RA conditions from day 0 to day 2. The data are shown as the mean ± S.D (n=3). Statistically significant differences, calculated through student-t tests, are indicated (*, P<0.05; **, P<0.01; ***, P<0.001).

(B) Transwell invasive assay for WT, *Smad3-/-* and *Smad3-/-* with *Rif1* shRNA ES cells under -2i-LIF+RA conditions. Scale bar equals to 200μm. Invasive colonies were counted for at least 3 fields individually. The data are shown as the mean ± S.D

(n=3). Statistically significant differences, calculated through student-t tests, are indicated (*, $P<0.05$; **, $P<0.01$; ***, $P<0.001$).

(C) Quantitative real-time PCR to examine the mRNA levels of *Mmp2* and *Mmp9* in WT ES cells and *Smad3-/-* ES cells transfected with control shRNA and *Rif1* shRNA plasmids. *Actin* was analyzed as an internal control. The data are shown as the mean ± S.D (n=3). Statistically significant differences, calculated through student-t tests, are indicated (*, $P<0.05$; **, $P<0.01$; ***, $P<0.001$).

(D) Images of hematoxylin/eosin stained section of WT ES cell, control *Smad3-/-* ES cell and *Rif1* shRNA transduced *Smad3-/-* ES cell formed teratomas. All three germ layer tissues can be observed.

Figure S7

(A) Phase contrast microscopy of mouse teratocarcinoma cell lines P19-1, P19-2 and F9. Scale bar equals to 200μm.

(B) Quantitative real-time PCR to examine the mRNA levels of *Pou5f1, Smad3* and *Rif1* in mouse ESC-formed teratoma cells *in vivo* (TC-1 and TC-2) and mouse embryonic teratocarcinoma cell lines P19 (P19-1 and P19-2) and F9. *Actin* was analyzed as an internal control. The data are shown as the mean ± S.D (n=3).

Table S1 Primers used in the study
